# Metagenomic Insight Into Patterns and Mechanism of Nitrogen Cycle During Biocrust Succession

**DOI:** 10.3389/fmicb.2021.633428

**Published:** 2021-03-17

**Authors:** Qiong Wang, Yingchun Han, Shubin Lan, Chunxiang Hu

**Affiliations:** ^1^Key Laboratory of Algal Biology, Institute of Hydrobiology, Chinese Academy of Sciences, Wuhan, China; ^2^University of Chinese Academy of Sciences, Beijing, China

**Keywords:** nitrogen cycle, biocrusts, succession, metagenome, GeoChip

## Abstract

The successional ecology of nitrogen cycling in biocrusts and the linkages to ecosystem processes remains unclear. To explore this, four successional stages of natural biocrust with five batches of repeated sampling and three developmental stages of simulated biocrust were studied using relative and absolute quantified multi-omics methods. A consistent pattern across all biocrust was found where ammonium assimilation, mineralization, dissimilatory nitrite to ammonium (DNiRA), and assimilatory nitrate to ammonium were abundant, while denitrification medium, N-fixation, and ammonia oxidation were low. Mathematic analysis showed that the nitrogen cycle in natural biocrust was driven by dissolved organic N and NO_3_^–^. pH and SO_4_^2–^ were the strongest variables affecting denitrification, while C:(N:P) was the strongest variable affecting N-fixation, DNiRA, nitrite oxidation, and dissimilatory nitrate to nitrite. Furthermore, N-fixation and DNiRA were closely related to elemental stoichiometry and redox balance, while assimilatory nitrite to ammonium (ANiRA) and mineralization were related to hydrological cycles. Together with the absolute quantification and network models, our results suggest that responsive ANiRA and mineralization decreased during biocrust succession; whereas central respiratory DNiRA, the final step of denitrification, and the complexity and interaction of the whole nitrogen cycle network increased. Therefore, our study stresses the changing environmental functions in the biocrust N-cycle, which are succession-dependent.

## Introduction

The nitrogen cycle is not only a major element cycle related to climate change but also a burgeoning field of research for the global materials cycle (Delgado-Baquerizo et al., 2013; Weber et al., 2015; Keren et al., 2020; Li et al., 2020). Particularly, our understanding of the nitrogen cycle due to breakthrough discoveries of new microorganisms and techniques has been remarkable (Reverey et al., 2016; Stein and Klotz, 2016; Kuypers et al., 2018; Zehr and Capone, 2020). The existence of new metabolic pathways is the result of the balance and distribution of nitrogen in ecosystems (Rütting et al., 2011) and directly related to the stability of ecosystem function (Isobe and Ohte, 2014). However, the distributional patterns of each pathway and their links to ecological processes remain unclear (Isobe and Ohte, 2014).

The known nitrogen cycle involves 9 inorganic redox states and more than 14 transformation pathways (Stein and Klotz, 2016; Kuypers et al., 2018), although they are often habitat dependent (Lüke et al., 2016; Stein and Klotz, 2016; Li et al., 2018; Wang et al., 2020). Our understanding of the nitrogen cycle remains mostly at the chemical and individual organism level: from the classical view including nitrogen fixation, nitrification and denitrification, to the classification of assimilation [ammonium assimilation, nitrogen fixation and assimilatory nitrate to ammonium (ANRA)] and dissimilation [mineralization, ammonium oxidation, nitrite oxidation, denitrification and dissimilatory nitrate reduction to ammonium (DNRA)] (Thamdrup, 2012), to the division of ammonification, nitrification, denitrification, anammox and NO_2_^–^-NO_3_^–^ interconversion (Stein and Klotz, 2016). At the community level, although inferences have been made related to ecological processes (Rütting et al., 2011; Isobe and Ohte, 2014), there is limited knowledge from the perspective of ecology because of unknown nitrogen abundance and correlations in the nitrogen transformation processes. Environmental factors such as redox potential (Eh), pH, NH_4_^+^, NO_3_^–^, and C:(N:P) can affect different transformation pathways; however, most studies focus on a single pathway (Pett-Ridge et al., 2006; Burgin and Hamilton, 2007; Rütting et al., 2011; Sgouridis et al., 2011; Bouskill et al., 2012; Jiang et al., 2015; Zheng et al., 2020), but how these factors drive overall patterns in N-cycle is poorly understood.

Biological soil crusts (biocrusts) are the biological-soil mosaic layers on dryland surface and consist of *Cyanobacteria*, lichens, mosses, algae, and heterotrophic organisms in varying proportions. Biocrusts have been recognized as an ideal model system for soil eco-function research, where different types of biocrusts represent different developmental and successional stages, which significantly affect the stability and eco-functions of the local soil ecosystem (Bowker et al., 2014; Ouyang and Hu, 2017). Similar to other soil types, N-cycling in biocrusts is susceptible to precipitation or water (Zaady, 2005; McCalley and Sparks, 2008; Gu and Riley, 2010; Delgado-Baquerizo et al., 2014) and biocrusts are often in a nitrogen-limited state under drought conditions (Hooper and Johnson, 1999; Schulz et al., 2013). In response to episodic water, the N-cycle in biocrusts occurs in pulses (Austin et al., 2004; Collins et al., 2008), with N_2_O, NO, and HONO emissions (Abed et al., 2013; Weber et al., 2015). Although measurements of process rates are limited and inconsistent, typically, nitrogen fixation and ammonia oxidation are higher (Zaady, 2005; Johnson et al., 2007; Strauss et al., 2011), but occasionally denitrification is stronger (Abed et al., 2013). It is likely that no matter the soil or biocrust, they all perform differently depending on the intensity and frequency of water events (Reverey et al., 2016), leading to the inconsistencies in different N-cycle studies. Biocrusts under different successional stages may have distinct microbial community structures and redox states, thus, the potential nitrogen transformation rates measured under similar experimental conditions are difficult to compare, and may not reflect biological reality.

The soil N-cycle is sensitive to water availability, whereas the patterns in metagenomic sequencing are relatively stable (Nelson et al., 2016). We speculate that the stable N-cycle genetic potential of different biocrusts can be obtained through repeated sampling, with distinguishable phenotypic and relatively stable eco-physiological characteristics of biocrusts (Grote et al., 2010; Lan et al., 2017; Maier et al., 2018). We can determine the relevance of different N pathways by coupling them with gene abundance relationships. Furthermore, the relationship between environmental variables and N pathways will help us understand the driving mechanisms of biocrust succession and relevant changes in ecological functions. In this study, four types of natural biocrusts representing successional stages with *Cyanobacteria* being replaced by lichens or moss, were repeatedly sampled, and three types of simulated biocrusts in which *Cyanobacteria* gradually become the dominant microorganism were also analyzed as controls, in order to explore the successional variability of N-cycle and its linkage mechanism to ecosystem functions. We hypothesized that the N-cycle which included diverse transformation processes in biocrusts is not a simple balance of nitrogen fixation and denitrification, in the environment of biocrusts with similar pH (Lan et al., 2015), those N-transformation processes will be significantly regulated by the variables closely related to redox condition, and the ability of the microbial community to coordinate the nitrogen input, loss, retention, recycle and utilization will improve with succession.

## Materials and Methods

### Sites Description and Sampling

Four types of natural biocrusts (algae crusts, A; cyanolichen crusts, C; chlorolichen crusts, G; and moss crusts, M) were collected in 2015, 2016, 2017, and 2018 from the Shapotou area located near the southeastern edge of the Tengger Desert (37° 32′ N, 105° 02′ E), China, by carefully sampling pieces of crusts in their natural thickness (4–15 mm; without the soil beneath) with a sharp shovel as described previously (Lan et al., 2015). Among them, algae crusts, lichen crusts and moss crusts represent the early, transitional, and later stages of succession, respectively, and the two types of lichen crusts have distinct survival strategies (Lange et al., 1998; Grote et al., 2010; Lan et al., 2017). Simulated crusts were cultured *in situ* in September and October 2017 at the east edge of the Hobq Desert (40° 21′ N, 109° 51′ E) (Ouyang et al., 2017). *Microcoleus* sp. isolated from biocrusts of Shapotou were inoculated onto the shifting sandy surface in September 2017, watered twice daily (08:00 and 18:00) during the first week only, and collected on the 11th, 35th, and 52nd day post-inoculation to represent early (Ae), middle (Am), and later (Al) stages of development, respectively. For 2015 natural biocrusts, the analysis of GeoChip and sequencing of 16S rRNA gene and N-cycle genes were completed in four replicates, while all other analyses (including metagenomics) were completed in triplicate. Natural biocrusts in 2016 and 2017 have only one replicate, but cyanolichen crusts included two types. Two batches of natural biocrusts were sampled in 2018 with four replicates for each type. For simulated biocrusts, there were four replicates for the three stages. A schematic of the experimental and analytical workflow is provided in Supplementary Figure S1.

### Soil Analyses and Characterization

Immediately after the samples reached the laboratory, the water content (WC) (Zhang et al., 2018) and texture (Olmstead et al., 1930) were measured. Electrical conductivity, pH, and Eh were measured in a soil suspension with a soil:water ratio of 1:5 (w/v). Total carbon, organic carbon and dissolved organic carbon (DOC) were determined by carbon analyzer (vario TOC, Elementar, Germany). Total nitrogen and total phosphorus content were analyzed using spectrophotometry (Lan et al., 2015). Biomass carbon was analyzed via chloroform fumigation (Vance et al., 1987), and remnant carbon was calculated by subtracting biomass carbon from organic carbon. The contents of CO_3_^2–^ and HCO_3_^–^ were measured by double indicator titrations, that of NO_3_^–^, NO_2_^–^, NH_4_^+^, PO_4_^3–^, Ca^2+^, and Mg^2+^ by ion chromatography. Dissolved organic nitrogen (DON) was calculated as the difference between total N and inorganic N (Zhou et al., 2020).

### DNA Extraction and qPCR

DNA was extracted using the PowerSoil DNA Isolation Kit (MO BIO Laboratories, Carlsbad, United States), and isolates were stored at −80°C. DNA was quantified and examined for purity with NanoDrop 2000c (Thermo Fisher Scientific, United States). Absolute copies of bacterial, fungal, archaeal, and cyanobacterial rRNA genes, and *nifH* and *nrfA* genes were quantified by qPCR (Applied Biosystems StepOnePlus^TM^ Real-Time PCR system). The qPCR conditions and primers are given in Supplementary Table S1. All the qPCR reactions were run in triplicate.

### Illumina MiSeq Sequencing of 16S rRNA Gene and N-Cycle Genes

Sequences associated with nitrogen fixation (*nifH*), denitrification (*nirK*), and bacterial 16S rRNA were amplified using the following primer pairs: *nifH*_F/*nifH*_R (Rosch et al., 2002), *nirK*_F1aCu/*nirK*_R3Cu (Throback et al., 2004), 338F/806R (Castrillo et al., 2017). DNA libraries were sequenced on the Illumina MiSeq platform with 2 × 250 pair-ends. For 16S rRNA analysis (Supplementary Table S2), sequences of chloroplasts were removed. All quality-filtered sequences were clustered into OTUs at 97% identity and randomly resampled to the depth of minimum number of sample sequences. Representative sequences of 16S rRNA gene were annotated taxonomically against the SILVA 138 database with a confidence cutoff at 0.7. Other sequences were classified by Blastn against the non-redundant protein database^1^.

### Metagenomic Sequencing and Assembly

Metagenomic libraries were sequenced on Illumina platforms (Supplementary Table S2). Raw sequence data quality was assessed using FastQC^2^, trimmed using Seqprep^3^, and high quality reads were extracted by filtering low-quality reads with “N” base, reads with < Q20, and short reads of < 50 bp using Sikle^4^. *De novo* assembly via de Brujin graph approach was performed using the multiple mixing assembly strategy with IDBA-UD (Peng et al., 2012) (k-mer = 47–97) and Newbler^5^. Contigs (≥300 bp) were analyzed for open reading frames (ORFs) prediction using MetaGene (Noguchi et al., 2006). Non-redundant gene catalog was constructed with predicted ORFs (≥100 bp) using CD-HIT (Li and Godzik, 2006) at 95% identity and 90% coverage. All high-quality reads were aligned (95% identity) against the gene catalog via SOAPaligner (Li et al., 2008) to obtain the gene abundance in each sample. The abundance of each unigene was normalized with gene length as previously described (Qin et al., 2012; Zheng et al., 2016).

### Metagenome Annotation

Taxonomic analysis was conducted by searching the putative amino acid sequences against NR databases (June 2018) using BLASTp (BLAST version 2.3.0; *E*-value ≤ 1e-5). To analyze the microbial nitrogen cycle, and minimize the influence of bryophytes whose genome size is much larger than microbes, the sequences that were “Bryopsida” at the class level were removed, and the remaining sequences were used for analysis of subsequent functional annotations using BLASTp against KEGG (version 59). The corresponding relationship between all the N-cycle associated genes and KEGG Orthology number is shown in Supplementary Table S3. Each gene combination or pathway was summed (multiple pathways performing the same conversion step) or averaged (multiple enzymes/subunits in the same conversion step) and normalized to 1,000,000 (Llorens-Mares et al., 2015).

### GeoChip Analysis

GeoChip 5.0 (180K) analysis was performed as described previously (He et al., 2007; Yang et al., 2013) with little modification. Probes were considered positive if their signals were detected in at least 2 of 4 replicate sets. The signal intensity of each gene or functional category was divided by the number of probes included in this gene or functional category to reduce the impact of the number of probes.

### Potential Activity of N-Cycle Pathway

Potential N fixation activity was measured by acetylene reduction assays (Belnap, 2002; Strauss et al., 2011). Biocrusts were evenly moistened with glucose-containing solution and incubated in a 100 mL serum bottle at 26°C for 8 h. Potential ammonia oxidation activity was determined according to methods described previously (Johnson et al., 2005; Strauss et al., 2011). Biocrusts were mixed with 1 mM (NH_4_)_2_SO_4_ and 75 mM NaClO_3_ and incubated on a shaker at 24°C for 10 h. The change in NO_2_^–^ concentration between 2 and 10 h was measured. The acetylene inhibition assay was used to determine the potential denitrification activity (Strauss et al., 2011). Biocrusts and sterile denitrification media (containing 100 mg/L KNO_3_ and 100 mg/L glucose) were mixed in a bottle, purged with nitrogen, and incubated at 24°C in the dark. The increase in N_2_O concentration after 4 and 8 h was measured.

### Statistical Analysis

The significant differences between various indicators in the samples were determined with SPSS 20.0 software (IBM, United States) using one-way analysis of variance (ANOVA) at *p* < 0.05. Non-metric multidimensional scaling analysis (NMDS) and redundancy analysis (RDA) were conducted using Canoco 5 (Šmilauer and Lepš, 2014). Analysis of similarity (ANOSIM) were conducted to test the statistical significance of differences between different types of biocrusts with *vegan* package in R, performing 10,000 permutations. Spearman’s correlation was calculated using *psych* package in R 3.4.4. Network diagrams were visualized with Cytoscape (Shannon et al., 2003). The co-occurrence network of the N-cycle key genes and high abundance genus (average abundance for each type in each batch above 0.01%, represent the top 833 of all 5,998 detected genus) was clustered with an edge-weighted spring-embedded algorithm with correlation coefficient r score. Each connection stands for a significant (*p* < 0.001) correlation. ModuLand as a Cytoscape plug-in (Kovacs et al., 2010) was used for network modularization, with a threshold of merging modules at 0.9. The network dissimilarity (β_w_) between different successional stages was determined using previously described methods (Koleff et al., 2003; Liu et al., 2019). Details of methods can be found in Supplementary Material.

## Results

### Microbial Community Structure

**FIGURE 1:**
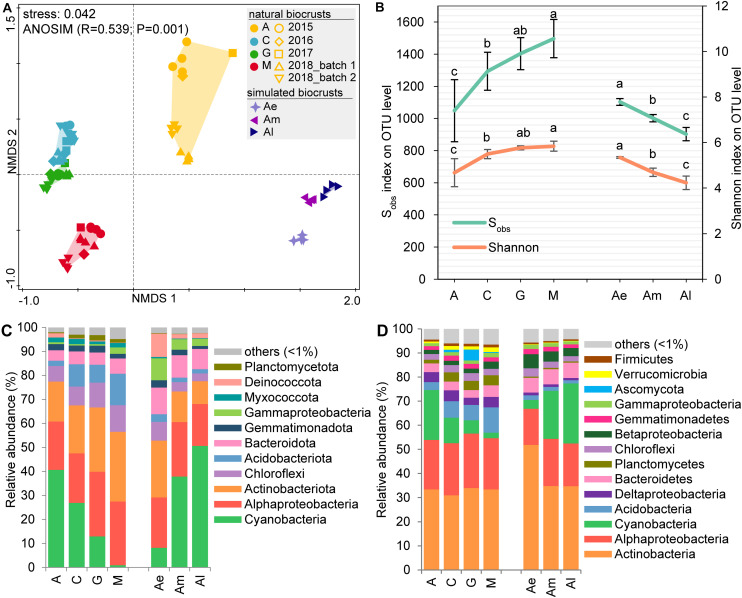
Community structure of biocrusts. **(A)** Non-metric multidimensional scaling (NMDS) combined with analysis of similarity (ANOSIM) for bacterial community dissimilarity based on 16S rRNA gene sequencing (OTU level). Colors represent different stages of biocrusts; shapes represent sampling batches. **(B)** Alpha diversity (S_obs_ and Shannon) of bacterial communities based on 16S rRNA amplicon data set compared across different stages of biocrusts. **(C)** Mean relative abundance of primary bacterial phyla (*Proteobacteria* in class) with 16S rRNA gene sequencing. **(D)** Taxonomic analyses based on the metagenomic data on phyla level (*Proteobacteria* in class). A, C, G, M represent four types of natural biocrusts at different succession stages. A, algae crusts, *n* = 13; C, cyanolichen crusts, *n* = 15; G, chlorolichen crusts, *n* = 13; M, moss crusts, *n* = 13. Ae, Am, and Al, early, middle, and later stage of simulated cyanobacterial crusts, respectively.

The NMDS analysis of 16S rRNA gene sequences (Figure 1A) at the OTU-level showed that bacterial community structure had significant differences among different successional stages of natural biocrusts and also different developmental stages of simulated biocrusts (ANOSIM, *R* = 0.539, *P* = 0.001). The diversity index (S_obs_ or Shannon) increased with the succession of natural biocrusts but decreased throughout the development of simulated biocrusts (Figure 1B). For community composition, in both 16S rRNA gene sequencing (Figure 1C) and metagenomic sequencing (Figure 1D), the top three most abundant taxa were *Cyanobacteria*, *Actinobacteria*, and *Alphaproteobacteria* in both early (A) and transitional stages (C, G) of natural biocrusts, as well as middle (Am) and late stages (Al) of simulated biocrusts. With the succession of natural biocrusts, the relative abundance of *Cyanobacteria* decreased, while *Acidobacteria* and *Gammaproteobacteria* increased, but the trend was reversed through the development of simulated biocrusts. Thus, the abundance of *Acidobacteria* was higher than that of *Cyanobacteria* in late stage (M) natural biocrusts, and the abundance of Bacteroides was higher than that of *Cyanobacteria* in early stage (Ae) of simulated biocrusts.

### The Abundance of N-Cycle Pathways

**FIGURE 2:**
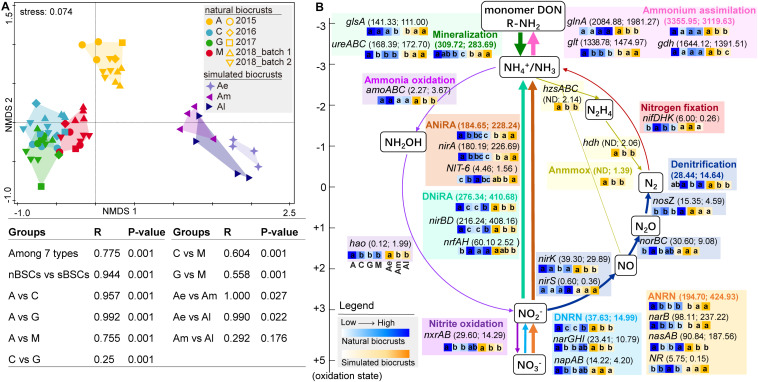
Nitrogen cycle based on metagenomic sequencing analysis. **(A)** NMDS and ANOSIM showing the significant difference of N-cycle for different stages of natural biocrusts (nBSCs) or simulated biocrusts (sBSCs) based on KEGG Orthology level. **(B)** Relative abundance of N-cycle genes or pathways. Different colors represent different nitrogen transformation pathways, and the values after pathways or genes are the mean relative abundance of nBSCs (5 batches) and sBSCs. Arrow width is proportional to the abundance of the nitrogen pathways. Heatmap from left to right indicate the change of pathways or genes with the succession of nBSCs (blue) or the development of sBSCs (yellow), and different letters represent significant differences within nBSCs or sBSCs (Tukey HSD; *p* < 0.05). Denitrification here indicates the transformation of NO_2_^–^ to N_2_. DNRN, dissimilatory nitrate to nitrite; DNiRA, dissimilatory nitrite to ammonium; ANRN, assimilatory nitrate to nitrite; ANiRA, assimilatory nitrite to ammonium; ND, not detected. DON, dissolved organic N.

From metagenomic sequencing data, pathways involved in the N-cycle can be indicated by relevant genes. Based on metagenomic sequences, NMDS and ANOSIM analyses showed that, the N-cycle was significantly different among different successional stages of natural biocrusts, early (Ae), and middle-late (Am, Al) simulated biocrusts (*R* = 0.775, *P* = 0.001) (Figure 2A). For nitrogen transformation processes in natural biocrusts (Figure 2B), the relative abundance of ammonium assimilation (*gdh*, *glnA*, *glt*) was the highest, followed by mineralization (*ureABC*, *gls*), DNiRA(*nirBD*, *nrfAH*) and ANRA (*nasAB*, *narB*, *NR*, *nirA*, *NIT-6*), while that of DNRN (*narGHI*, *napAB*), nitrite oxidation (*nxrAB*) and denitrification (*nirK*, *nirS*, *norBC*, *nosZ*) were lower; and nitrogen fixation (*nifDHK*) and ammonia oxidation (*amoABC*) were the lowest. The abundance of genes for denitrification (*nirK*, *nirS*, *norBC*, *nosZ*) decreased along with the reaction sequences (Figure 2B). Except for cyanolichen crusts which have a special survival strategy, with the succession of natural biocrusts, the abundances of *nrfAH* and *nosZ* increased, while other genes such as *glt*, *ureABC*, *gls*, *amoABC*, *hao*, *narB*, *nirA*, *nirBD* showed a decreasing trend. The pattern of N-cycle in simulated biocrusts was similar to that of natural biocrusts, with a slightly higher relative abundance of anammox (*hzsABC*, *hdh*), *nirBD* in DNiRA, and *nasAB*, *narB* and *nirA* in ANRA. Along with the development of simulated biocrusts, the abundances of some genes such as *narB*, *nirA*, *NR*, *ureABC*, *gls* and *glt* increased, while the abundances of some other genes such as *amoABC*, *hao*, *nxrAB*, *nirBD*, *nrfAH*, *nirK*, *nosZ*, *narGHI*, *glnA*, and gdh decreased.

**FIGURE 3:**
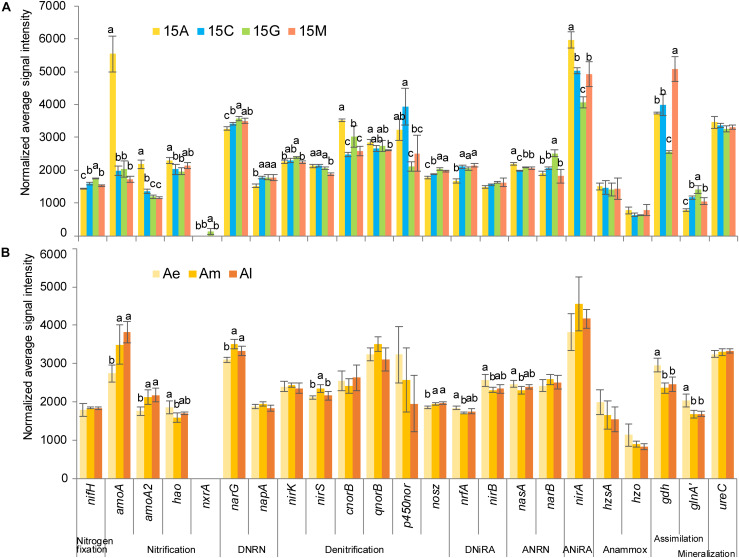
Normalized average signal intensity of key genes in nitrogen cycle for natural biocrusts in 2015 **(A)** and simulated biocrusts **(B)**. Error bars represent standard errors, and different letters represent significant difference (Tukey HSD; *p* < 0.05) within natural biocrusts (*n* = 4) or simulated biocrusts (*n* = 4). amoA2, glnA’ represent the gene names of amoa_quasi and glnA_fungi in GeoChip, respectively.

From the absolute quantitative analysis using GeoChip (Figure 3), the normalized average signal intensity of *amoA* (*amoA*, *amoA2*), *nirA* and *ureC* gradually decreased along the succession of natural biocrusts, while *nrfA* and *nosZ* showed the opposite trend. With the development of simulated biocrusts, an increased signal intensity was observed for genes *nirA*, *narB*, and *ureC*, while a decreasing trend was observed for *nrfA* and *p450nor*. In addition, genes involved in anammox (*hzsA* and *hzo*) were detected both in natural biocrusts and simulated biocrusts.

**FIGURE 4:**
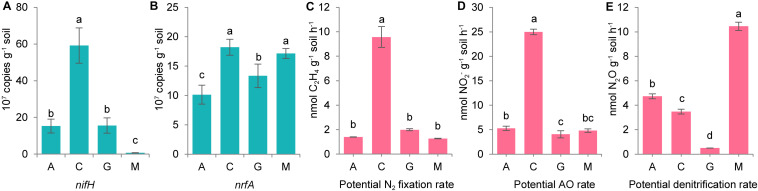
The gene copies of *nifH* **(A)** and *nrfA* **(B)**, and the potential rate of classical nitrogen cycle including N_2_ fixation **(C)**, ammonia oxidation (AO, **D)**, and denitrification **(E)** for natural biocrusts in 2015. Error bars represent standard errors, and different letters represent significant difference (Tukey HSD; *p* < 0.05) within natural biocrusts (*n* = 4) or simulated biocrusts (*n* = 4).

From quantitative PCR analysis (Figures 4A,B), we confirmed that gene copies of *nifH* and *nrfA* were highest in cyanolichen crusts. Potential nitrogen fixation and ammonia oxidation rates were both highest in cyanolichen crusts, and potential denitrification rates were highest in moss crusts (Figures 4C–E).

### The Potential Microbial Contribution to N-Cycle

**FIGURE 5:**
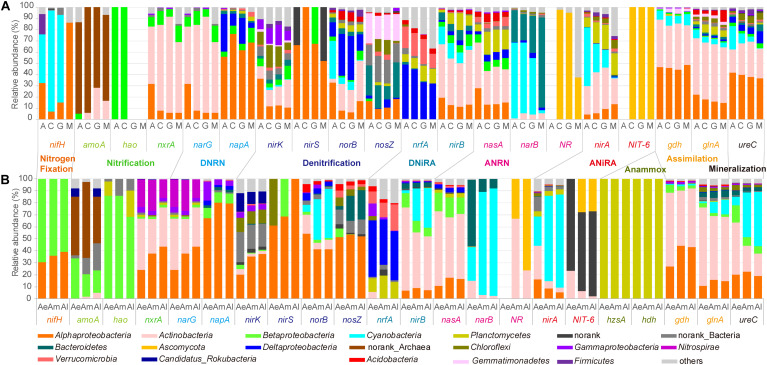
Relative abundance of the microbial taxa related to each key gene of nitrogen cycle for natural biocrusts in 2015 **(A)** and simulated biocrusts **(B)**.

In natural biocrusts (Figure 5A), marker gene related to nitrogen fixation (*nifH*) mostly belonged to *Cyanobacteria* in early (A) and transitional stages (C, G), while *Alphaproteobacteria* dominated later stages (M). Main taxa involved in ammonium assimilation (*gdh*, *glnA*) and mineralization (*ureC*) were *Alphaproteobacteria* and *Actinobacteria*. Ammonia oxidation (*amoA*) was mostly carried out by Archaea. While *Alpha*-, *Beta*-, *Delta*-*proteobacteria*, and *Actinobacteria* were the main taxa contributing to interconversion of NO_2_^–^-NO_3_^–^ (*nxrA*, *narG*, *napA*); *Alpha*-, *Delta*-*proteobacteria*, *Verrucomicrobia*, *Actinobacteria*, *Cyanobacteria*, and *Planctomycetes* were the main potential taxa involved in DNiRA (*nrfA*, *nirB*). For both assimilatory nitrate to nitrite (ANRN) (*nasA*, *narB*, *NR*) and ANiRA (*nirA*, *NIT-6*), microbes such as *Actinobacteria*, *Cyanobacteria*, *Ascomycota*, *Planctomycetes*, and *Bacteroidetes* were found. Potential denitrifiers were different depending on the reaction steps, but mainly belonging to *Proteobacteria* (*Alpha*-, *Gamma*-, *Delta*-), *Actinobacteria*, *Cyanobacteria*, *Chloroflexi*, *Gemmatimonadetes*, or *Bacteroidetes*.

In simulated biocrusts (Figure 5B), sequences matching to N-cycle were identified as similar organisms to those in natural biocrusts with a few differences: (1) potential nitrogen fixers were mainly *Alpha*- and *Beta*-*proteobacteria*; (2) as well as Archaea, *Betaproteobacteria* was also important for ammonia oxidation; (3) there were more *Nitrospirae* containing gene *nxrA* or *narG*; (4) *Alphaproteobacteria* were the most important bacteria involved in each step of denitrification; (5) Anammox always showed up in *Planctomycetes*.

### Co-occurrence Network of Microbial Community and the N-Cycle

**FIGURE 6:**
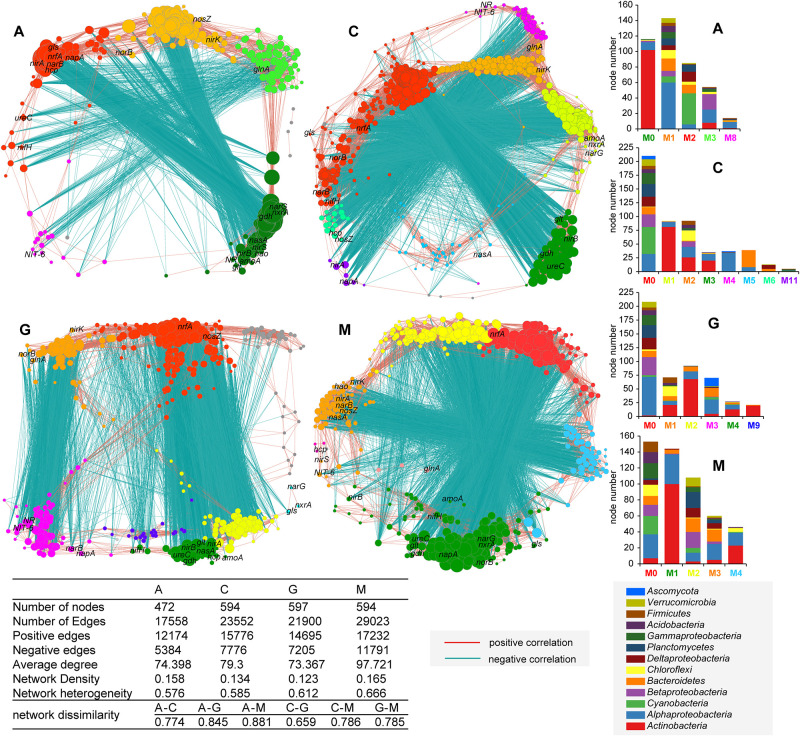
Co-occurrence network of the N-cycle key genes and high abundance of species for different successional stages of natural biocrusts (*n* = 13 for each stage). The colors of nodes represent different module, which are in line with the colors of abscissa label of histogram on the right (M stands for “module”). The community compositions of modules that contain few nodes are not shown in histogram. The size of the nodes corresponds to the degree of nodes. The connection stands for a significant spearman correlation (*p* < 0.001).

Based on the correlation (*p* < 0.001) of highly abundant genera and N-cycle marker genes, different networks were drawn for the four successional stages of natural biocrusts (Figure 6). Pairwise dissimilarity increased throughout biocrust succession. In the early (A) and transitional stages (C, G), the number of positive and negative edges, average degree, and network heterogeneity were lower; while they were higher in the late stages (M). For algae crusts (Figure 6; A), genes related to denitrification (*nirK*, *norB*, *nosZ*) and genera from several phylum formed a functionally independent module, as did the ammonium assimilation gene (*glnA*) and genera mainly from *Proteobacteria*; communities dominated by *Cyanobacteria* associated with genes such as *nifH* and *nrfA*. For cyanolichen crusts (Figure 6; C), there were more functional modules, such as the group dominated by *Cyanobacteria* and *Alphaproteobacteria* and associated with genes such as *nifH*, *nrfA*; the group coupled *Alphaproteobacteria* with *NR* and *NIT-6*. For chlorolichen crusts (Figure 6; G), there were also more modules, such as genera mainly from *Alphaproteobacteria* clustered with *nrfA* and *nosZ*; *nifH*, *nirB* and many other genes, clustered together. Communities dominated by *Ascomycota* and *Alphaproteobacteria* grouped together with *NR*, *NIT-6*. For moss crusts (Figure 6; M), *nrfA* and genera from several phyla formed the largest independent module. Another group was dominated by *Actinobacteria* and associated with genes such as *nifH*, *nirB*. With the succession of natural biocrusts, network complexity increased, and the associated functions such as denitrification, DNiRA, *glnA*, and *nifH* showed obvious shifts.

### Relevance of Pathways and Influence of Environmental Variables

**FIGURE 7:**
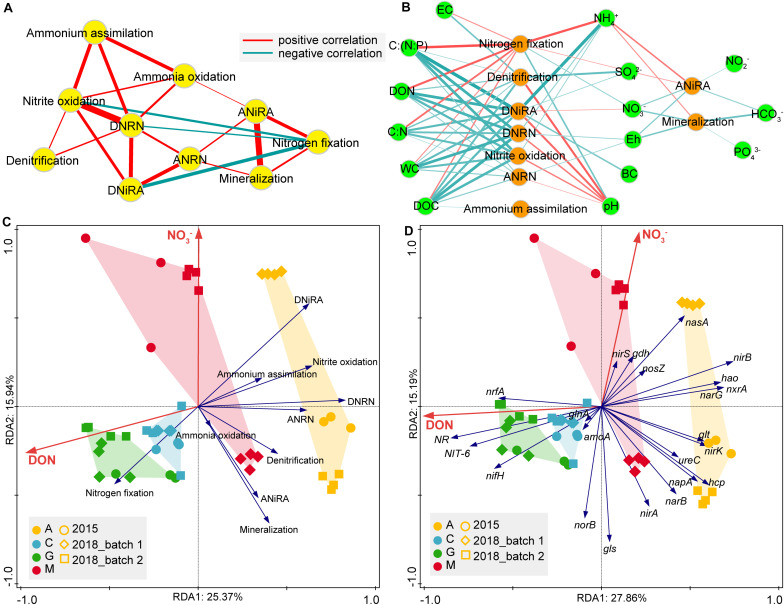
The spearman correlations between N-transformation pathways **(A)**, as well as between environmental factors and N-transformation pathways (**B**), were depicted using Cytoscape. Environmental factors that shaped nitrogen cycle significantly (*p* < 0.05) are shown on pathway level **(C)** or gene level **(D)** for natural biocrusts with forward selection of RDA. For network **(A,B)**, the connection stands for a significant spearman correlation (*p* < 0.001), and the edge width is proportional to the magnitude of correlation coefficient (|r|). EC, electrical conductivity; WC, water content; Eh, redox potential; DON, dissolved organic nitrogen; DOC, dissolved organic carbon; BC, biomass carbon.

To reveal the relevance of N-cycle pathways, correlation networks (Figure 7A) were used. We found a strong positive correlation between DNRA and nitrite oxidation (*R* = 0.896, *P* < 0.001). A similar relationship was also observed between ANiRA and mineralization (*R* = 0.864, *P* < 0.001). As the hub of the network, DNRN and nitrite oxidation with the highest degree were significantly positively correlated with the most pathways. ANiRA and mineralization were positively correlated with nitrogen fixation and ANRN; while nitrogen fixation was negatively correlated with DNiRA, nitrite oxidation, and DNRN.

For the environmental factors that influenced each N-cycle pathway, the correlation network (Figure 7B) showed that, NH_4_^+^ was significantly and positively correlated with nitrogen fixation, ANiRA, and mineralization. In contrast, the remaining variables [C:(N:P), DON, C:N, WC, and DOC] were positively correlated with nitrogen fixation, but negatively correlated to the other six intermediate pathways (denitrification, DNiRA, DNRN, nitrite oxidation, ANRN and ammonium assimilation). Compared with other variables, C:(N:P) had the strongest correlation with DNiRA, followed by DNRN, nitrite oxidation, and ANRN, while pH was negatively correlated with nitrogen fixation and positively correlated with denitrification, followed by DNiRA, DNRN, nitrite oxidation, and ANRN. In addition, Eh, NO_3_^–^, and HCO_3_^–^ were negatively correlated with both ANiRA and mineralization, while SO_4_^2–^ was most strongly negatively correlated with denitrification. In short, both pH and SO_4_^2–^ were the most important variables affecting denitrification. However, pH and C:(N:P) were not only the most important variables affecting nitrogen fixation but also the most important variables affecting DNiRA, nitrite oxidation, and DNRN.

Furthermore, to explore what environmental factors shaped the whole N-cycle function in natural biocrusts, RDA was used and showed that DON and NO_3_^–^ were significant variables explaining the variance of the N-cycle in natural biocrusts (*p* < 0.05), either at the pathway or gene level (Figures 7C,D). DON was positively correlated with the relative abundance of *nrfA*, but negatively correlated to some other genes, such as *nirB*, *nxrA*, and *narG*; while NO_3_^–^ was positively correlated with the relative abundance of *nasA* and negatively correlated with the relative abundances of genes, such as *nirA* and *norB* (Figure 7D).

## Discussion

In our study, we investigated microbial community structure using 16S rRNA (Figure 1C), *nifH* or *nirK* (Supplementary Figure S2) gene amplicon sequencing and metagenomic sequencing (Figures 1D, 5A). Community structures obtained from both methods were similar, although we cannot rule out issues caused by the limitation of primers and unknown microbes. We analyzed 5 batches of data from 4 different years (Figure 2) and found that the relative abundances of genes involved in denitrification decreased as reaction steps proceeded (*nirK* > *norBC* > *nosZ*), further supporting decreased growth yields per molar substrate (Koike and Hattori, 1975), as well as increases in oxygen sensitivity of denitrification related enzyme activities (Kraft et al., 2011). Although changing sequencing platforms and multiple batches may have biased our results, the differences between different successional stages are quite large (Figure 2A), confirming the stability and reliability of our outcomes. Thus, the genetic characteristics of the four types of natural biocrusts in the present study represent the different successional stages very well and fully meet our experimental expectations. Furthermore, in both natural and simulated biocrusts, we confirmed from the metagenomic data that the basic pattern of the N-cycle was that ammonium assimilation, mineralization, DNRA, and ANRA were higher, while denitrification and nitrite oxidation, nitrogen fixation, and ammonia oxidation were lower, with anammox being the lowest. This is consistent with the general pattern that ammonium assimilation is common, and nitrogen fixation and ammonia oxidation are less common in most soils (Varin et al., 2010; Nelson et al., 2015, 2016; Souza et al., 2015; Tu et al., 2017), but it is notably different from that in sandy saline soils (Ren et al., 2018), marine oxygen minimum zones (Ganesh et al., 2014), and sediment (McTigue et al., 2016; Rasigraf et al., 2017) with abundant denitrification. The organic-rich biocrusts dwell on the soil surface, in which the material cycle is mainly driven by light energy. In organic-poor deep seas (Rasigraf et al., 2017) or stratified lake bottoms (Llorens-Mares et al., 2015), as well as water environments rich in nitrate (Lüke et al., 2016; Li et al., 2018; Keren et al., 2020), the material cycle is mainly driven by chemical energy. We speculate that the difference in material cycling pattern is determined by the most abundant electron donors and receptors available in the different habitats.

From the perspective of ecological function, the nitrogen cycle in biocrusts is also as expected. In the DON-rich biocrusts, nitrogen fixation would not be abundant because of the high energy investment, polymetallic cofactor, and strict anaerobic conditions. In line with nitrogen fixation, nitrification is not expected to be high, especially NH_4_^+^-N, as the inorganic nitrogen form is preferred by dominant organisms *Cyanobacteria*, lichens, and mosses (Lin and Stewart, 1997; Dahlman et al., 2004; Muro-Pastor et al., 2005; Liu et al., 2013) and is volatile under high temperature and strong radiation. Except for the lack of NH_4_^+^-N substrate, hypoxia (Johnson et al., 2005; Ouyang and Hu, 2017) and other factors also inhibit nitrification (Castillo-Monroy et al., 2010). Denitrification with medium abundance is characterized by multiples steps, substrates, and products (N_2_/N_2_O/NO). However, biocrusts are rich in organic matter and have changeable redox states, which would increase the probability of denitrification (Tiedje et al., 1982). Nitrite oxidation is limited by low NO_2_^–^ in biocrusts, but it is a necessary process to convert inorganic nitrogen to the highest oxidation state and balance the redox potential, which helps explain why nitrite oxidation is in moderate abundance in biocrusts. Finally, ammonium assimilation, mineralization, and nitrate ammonification (DNRA and ANRA) are abundant in biocrusts because all of them play important roles in biocrust development. Among them, ammonium assimilation is an indispensable potential for the growth of most organisms. Mineralization is not only an important source of nutrients for organismal growth but also a necessary process during species replacement (Reverey et al., 2016). ANRA has the function of supplying NH_4_^+^-N at low cost, and DNRA has the capacity of energy yield (Cole and Brown, 1980). Both ANRA and DNRA use a two-step reaction to transform nitrogen from the highest oxidation state to the lowest reduction state and increase the nitrogen retention in a given ecosystem (Tiedje, 1988). Roughly, ANRA and DNRA match the abundances of ammonium assimilation and mineralization and eventually reduce the nitrogen loss in biocrusts, particularly when they frequently experience dry-wet cycles in the field. Therefore, the patterns of N-cycle in biocrusts can be regarded as an ideal ecological strategy with low energy cost and high nitrogen retention. RDA analysis (Figures 7C,D) suggest that the nitrogen cycling pattern of biocrusts is mainly driven by DON and NO_3_^–^. The DON is a highly reducing electron donor, and NO_3_^–^ is a highly oxidizing electron acceptor. Their influence is a multidimensional control of redox potential from high to low or vice versa.

Among the correlation of pathways (Figure 7A) in biocrusts, nitrite oxidation and DNRN occupy a hub position in the network with highest degree, and key genes have close genetic relationships (Kirstein and Bock, 1993; Simon and Klotz, 2013). The two processes are related to the substrates competing via denitrification, DNRN and ANRN, and are mainly affected by C:(N:P) and DOC (Figure 7), which supports the proposal of Stein and Klotz (2016) to define NO_2_^–^-NO_3_^–^ interconversion as an independent nitrogen-transformation flow. Compared with DNRN, ANRN had a much higher relative abundance (6x). Coupled with similar microbial communities between NO_3_^–^ transportation, assimilation, and ammonium assimilation (Figure 5 and Supplementary Figure S3), our results suggest DNRN is the true hub of nitrogen-transformation network. ANiRA and mineralization (Figure 7) are mainly affected by the characteristic variables (NH_4_^+^, Eh, NO_3_^–^) during hydrological changes (Reverey et al., 2016), while the nutrition strategies are significantly different among the potential bacteria (*Cyanobacteria*, *Proteobacteria*, *Chloroflexi*, *Planctomycetes*, and *Ascomycota*). Furthermore, the replacement of ecosystems is often accompanied by both decomposition and synthesis of organic matters caused by mineralization (Reverey et al., 2016), all of which indicate that these two processes closely respond to frequent dry-wet changes. Finally, DNiRA is a more advantageous process than denitrification under high C:NO_3_^–^ (Tiedje et al., 1982; Rütting et al., 2011). The *nmo* gene (encoding nitronate monooxygenase) in biocrust is comparable to *nirB* which may be employed for supplying NO_2_^–^ (Li et al., 2018). DNiRA and nitrogen fixation are strongly negatively correlated, and they are mainly affected by the opposite effects of NH_4_^+^, C:(N:P), DON and NO_3_^–^. NH_4_^+^ and C:(N:P) are subjected to stoichiometric constraints, especially for NH_4_^+^-N. And, DON and NO_3_^–^ are regulated by redox state. Therefore, nitrite oxidation and DNRN are the hubs connecting N-cycle pathways, DNiRA and nitrogen fixation are the most important ways to maintain the balance of elemental stoichiometry and redox states, while ANiRA and mineralization are processes that closely respond to dry-wet cycles.

Environmental variables such as Eh, pH, NH_4_^+^, NO_3_^–^, C:(N:P) influence N-cycle pathways (Pett-Ridge et al., 2006; Burgin and Hamilton, 2007; Rütting et al., 2011; Sgouridis et al., 2011; Bouskill et al., 2012; Jiang et al., 2015; Zheng et al., 2020). In this study, we found that pH played the opposite role in the N-cycle compared to many other variables (C:(N:P), NH_4_^+^, DON, SO_4_^2–^, C:N, WC, DOC). The effects of pH and C:(N:P) were the most significant, with pH negatively correlated to nitrogen fixation and positively correlated to denitrification. Different diazotrophic taxa respond differentially to soil pH (Wang et al., 2017), and free-living N fixers prefer neutral pH (Limmer and Drake, 1996), thus leading pH negatively correlate with N fixation in the slightly alkaline biocrust (pH 7.43–8.09), which is consistent with previous studies (Keshri et al., 2015; Wang et al., 2018a). The significant effects of C:(N:P) and C:N on nitrogen fixation were similar to those in forest succession, although single nutritional factors such as DON and SO_4_^2–^ did not affect nitrogen fixation significantly in forest ecosystems (Zheng et al., 2020). The increase in soil pH would solubilize organic matter and increase denitrification potential (Anderson et al., 2018). The negative correlation of SO_4_^2–^ and denitrification have been found previously (Wang et al., 2018b), for the reduction of SO_4_^2–^ can enhance DNRA and inhibit denitrification (Tiedje, 1988). More interestingly, the negative correlation of C:(N:P) with DNiRA, nitrite oxidation, DNRN, and ANRN decreased sequentially, and the positive correlation of pH with the above four pathways also decreased sequentially. This order is consistent with the order of thermodynamics-driven redox reactions (Falkowski et al., 2008; Ettwig et al., 2010), and, thus, we speculate that these four are the central pathways of N-cycle in biocrusts. Many variables significantly affect multiple pathways, but the key variables affecting the main pathways are different, in which pH and SO_4_^2–^ are the strongest variables affecting denitrification, and C:(N:P) is the strongest variable affecting nitrogen fixation, DNiRA, nitrite oxidation, and DNRN.

In order to further improve the reliability of metagenomic sequencing results (Figure 2 and Supplementary Figure S4) and compare gene abundances between different successional stages, we adopted the same result as GeoChip (Figure 3), qPCR, or potential rate (Figure 4). It has been recognized that acetylene reduction assay has limitation to examine potential N fixation rate such as differences in solubility of acetylene and N_2_ in water, adsorption of acetylene on soil colloids and toxicity to microbes, and ^15^N stable isotopes should be used instead (Barger et al., 2016). In this way, with the succession of natural biocrusts, the increase of *nrfA* and *nosZ* was associated with the increase of *Deltaproteobacteria*, *Verrucomicrobia*, *Gemmatimonadetes* (Figure 5); the decrease of *nirA* and *ureC* was related to the decrease of *Cyanobacteria* and the increase of *Bacteroidetes*, *Alphaproteobacteria* and *Actinobacteria* (Figure 5). However, with the development of simulated biocrusts, the increase of *nirA* and *ureC* was consistent with an increase in *Cyanobacteria*; the decrease of *nrfA* was associated with a decrease in weakly resistant bacteria such as *Deltaproteobacteria* and *Acidobacteria*. The changes of *nrfA* and *nirA* may be related to the abundance of electron donors and receptors under the influence of DON and NO_3_^–^ (Figure 7). Therefore, along with the succession of biocrusts, the changes of N-cycle follow the direction that ANiRA and mineralization gradually decreasing, respiratory DNiRA increasing, and more thorough denitrification, which indicated the improvement of nitrogen balance ability.

Our co-occurence network analysis (Figure 6) showed that the complexity and interaction degree between microbes and N-cycle pathways were significantly increased with succession of biocrusts, which supports the proposal of enhancement of microbial interactions that promote ecological succession (Datta et al., 2016) and fully displays ecosystem stability and functional enhancement of the N-cycle (Gravel et al., 2016). From the perspective of coupled relationships between species and genes, there were several obvious functionally independent groups, including two large groups in the early stage (A) coupled with denitrification, or ammonium assimilation (*glnA*) with higher affinity, and a group coupled with *nrfA* in the later stage (M), which is consistent with the harsher conditions of early stage (Geisseler et al., 2010; McTigue et al., 2016) and more nitrogen retention with increased organic matter in later stage (Moreno-Vivian et al., 1999; Rütting et al., 2011). The assimilation nitrate reduction group in the transitional stage (C, G) suggests that competition for nitrogen resources between fungi and bacteria exists in lichen crusts, which is likely accomplished by the process of nitrate reduction. However, *nifH* was clustered with *nrfA* in algae crusts and cyanolichen crusts, and *nirB* in chlorolichen crusts and moss crusts (Figure 6). Our data indicate that the functional groups and transformation processes of regulating nitrogen fixation are mainly the coordinated effects of *Cyanobacteria* and respiratory DNiRA function in the early (A) and early-middle stages (C), but the result of *Actinobacteria* and fermentive DNiRA in middle-late (G) and later stages (M).

As biocrusts in our study have adapted to dry-wet cycles for a long time, their functional microbial communities should be the result of long-term adaptation. In early natural biocrusts, nitrogen-fixing organisms were mainly dominated by *Cyanobacteria* (Yeager et al., 2012; Wang et al., 2016), while *Proteobacteria* played an important role in other biocrusts where nitrogen-fixing *Cyanobacteria* were not abundant (Pepe-Ranney et al., 2016). For aerobic ammonia oxidation, except for the reported *Thaumarchaeota* and *Betaproteobacteria* (Marusenko et al., 2013; Stein and Klotz, 2016), the proportion of unknown archaea in our study was also high. Microbes contributing to NO_2_^–^ and NO_3_^–^ interconversion were mainly *Proteobacteria* (*Alpha*-, *Beta*-, *Delta*-) and *Actinobacteria* (Alcantara-Hernandez et al., 2009; Daims et al., 2016), and *Nitrospirae* contributed more to simulated biocrusts. Compared with the microbes involved in DNiRA (Seitz and Cypionka, 1986; Guo et al., 2016; Cerqueira et al., 2018; Li et al., 2018; Keren et al., 2020) or ANRA (Nelson et al., 2015; Li et al., 2018; Ren et al., 2018) in other environments, there were more *Actinobacteria*, *Cyanobacteria*, and *Verrucomicrobia* in biocrusts associated with DNiRA, and more *Cyanobacteria*, *Planctomycetes*, and *Ascomycota* associated with ANRA. In denitrification, in addition to the reported *Proteobacteria*, *Bacteroidetes*, and *Chloroflexi* (Saarenheimo et al., 2015; Guo et al., 2016; Ren et al., 2018), there were abundant *Actinobacteria* (*nirK*, *norB*), *Cyanobacteria* (*norB*), and *Gemmatimonadetes* (*nosZ*) involved in the biocrusts. Overall, *Alphaproteobacteria*, *Actinobacteria*, and *Cyanobacteria* have the highest nitrogen transformation potential, while microbes that contained genes such as *amoA*, *nosZ*, and *nirK* were a higher proportion of unknown taxa.

From the perspective of physiology and ecology, the cyanolichen crust (C) being dominated by *Collema* in this study is a classic adaptive balance strategy for rapid biosynthesis under high temperature after sufficient rainfall (Lange et al., 1998; Grote et al., 2010). However, a deficit in material accumulation will occur when only a small amount of water is supplied (Ouyang et al., 2017). The other three types of biocrusts displayed common adaptations to water pulse (Lan et al., 2017; Ouyang et al., 2017). From our metagenomic analyses, the abundances of *nifDHK*, *nrfAH*, *amoABC*, and *norBC* were extremely high in the cyanolichen crusts, and gene copies of *nifH*, *nrfA*, and potential rate of nitrogen fixation and ammonia oxidation were also the highest. The only lower production of N_2_O in the cyanolichen crusts was related to the low DNRN and low concentration of NO_3_^–^ and NO_2_^–^ (Supplementary Table S4). This suggests that the difference in nitrogen transformation rate is only comparable when the samples have similar redox background, and the biocrusts with special survival strategies are not suitable for the comparison, which may be the reason for the divergences in the results from previous studies on potential nitrogen-transformation rates.

## Conclusion

This study conducted repeated sampling and metagenomic sequencing throughout 4 years and points toward the overall patterns and successional variation of N-cycle, the microbial contribution, the driving mechanism of environmental variables, the relevance between N-transformation pathways and between N-cycle and ecosystem functions. Our results highlight the consistent N-cycle pattern of low energy cost and high nitrogen retention ability in biocrusts, and an obvious successional variability of decreased responsive ANiRA and mineralization, increased respiratory DNiRA, and more thorough denitrification. Our results demonstrate that N-cycle in biocrusts is driven by DON and NO_3_^–^, which indicates a multidimensional control of redox potential. The linkage of N-cycle processes and the relevance with environmental variables show that nitrogen fixation and DNiRA are the most important pathways to maintain the balance of elemental stoichiometry and redox states, while ANiRA and mineralization are processes responding closely to the wet-dry cycles. In addition, our study suggests that the coordination of DNiRA and nitrogen fixation is crucial for N-cycle, and the role of two types of DNiRA changes with succession. This study indicated that biocrusts were an ideal natural model to research the function and succession of microbial ecosystems with metagenomic methods.

## Data Availability Statement

The datasets presented in this study can be found in online repositories. The name of the repository and accession numbers can be found below: National Center for Biotechnology (NCBI), https://www.ncbi.nlm.nih.gov/, PRJNA562239, PRJNA548650, PRJNA646196, PRJNA647185, PRJNA544139, PRJNA321565, PRJNA647637, PRJNA647192, PRJNA647699, PRJNA543295, PRJNA636669, and PRJNA636680.

## Author Contributions

CH designed the research and aided in data analysis. QW performed major experiments, carried out the analysis, prepared most figures. YH carried out parts of experiments of 2017 simulated biocrusts. CH and QW wrote this manuscript. SL provided advice and critically reviewed the manuscript. All authors read and approved the final manuscript.

## Conflict of Interest

The authors declare that the research was conducted in the absence of any commercial or financial relationships that could be construed as a potential conflict of interest.

## Footnotes

1 https://blast.ncbi.nlm.nih.gov/Blast.cgi

2 http://www.bioinformatics.babraham.ac.uk/projects/fastqc/

3 https://github.com/jstjohn/SeqPrep

4 https://github.com/najoshi/sickle

5 https://ngs.csr.uky.edu/Newbler

## Supplementary Material

Supplementary Figure 1 Schematic of the experimental and analytical workflow. A, C, G, M represent four types of natural biocrusts at different succession stages. A: algae crusts, C: cyanolichen crusts, G: chlorolichen crusts, M: moss crusts. Ae, Am, and Al represent early, middle, and later stage of simulated cyanobacterial crusts, respectively.

Supplementary Figure 2 Phylogeny of *nifH* **(A)** and *nirK* **(B)** genes from natural biocrusts in 2015. The heatmap and percentage from left to right are responding to the four types of natural biocrusts: algae crusts, cyanolichen crusts, chlorolichen crusts, and moss crusts, respectively. The evolutionary history was inferred using the Neighbor-Joining method in MEGA7.

Supplementary Figure 3 The potential microbial community involved in the transport of nitrate and nitrite in natural biocrusts in 2015. *NRT*, corresponding to K02575, encoding nitrate/nitrite transporter; *nrtA*, corresponding to K15576, encoding nitrate/nitrite transport system substrate-binding protein. A: algae crusts, C: cyanolichen crusts, G: chlorolichen crusts, M: moss crusts.

Supplementary Figure 4 The relative abundance of N-cycle pathways **(A)** or genes **(B)** based on metagenomic analysis in the natural biocrusts (5 batches) and simulated biocrusts.

Supplementary Table 1 The qPCR primers and conditions used in this paper.

Supplementary Table 2 Assembly and annotation statistics of metagenome and 16S rRNA gene sequences (ORF, open reading frame; QC, quality control).

Supplementary Table 3 The corresponding relationship of N-cycle pathways, genes and KEGG Orthology (KO) number.

Supplementary Table 4 The properties of biocrusts. Data are reported as average ± SD. Values with different letters are significantly different at 0.05 level within natural biocrusts (*n* = 3) or simulated biocrusts (*n* = 4). WC: water content; EC: electrical conductivity; Eh: redox potential; TC: total carbon; TOC: total organic carbon; BC: biomass carbon; RC: remnant carbon; TN: total nitrogen; TP: total phosphorus; DON: dissolved organic nitrogen. ND: not detected; NC: not collected.

## References

[B1] Abed R. M. Lam P. de Beer D. Stief P. (2013). High rates of denitrification and nitrous oxide emission in arid biological soil crusts from the Sultanate of Oman. *ISME J.* 7 1862–1875. 10.1038/ismej.2013.55 23575368 PMC3749496

[B2] Alcantara-Hernandez R. J. Valenzuela-Encinas C. Marsch R. Dendooven L. (2009). Respiratory and dissimilatory nitrate-reducing communities from an extreme saline alkaline soil of the former lake Texcoco (Mexico). *Extremophiles* 13 169–178. 10.1007/s00792-008-0207-1 19050822

[B3] Anderson C. R. Peterson M. E. Frampton R. A. Bulman S. R. Keenan S. Curtin D. (2018). Rapid increases in soil pH solubilise organic matter, dramatically increase denitrification potential and strongly stimulate microorganisms from the Firmicutes phylum. *PeerJ* 6:e6090. 10.7717/peerj.6090 30581677 PMC6295159

[B4] Austin A. T. Yahdjian L. Stark J. M. Belnap J. Porporato A. Norton U. (2004). Water pulses and biogeochemical cycles in arid and semiarid ecosystems. *Oecologia* 141 221–235. 10.1007/s00442-004-1519-1 14986096

[B5] Barger N. N. Weber B. Garcia-Pichel F. Zaady E. Belnap J. (2016). “Patterns and Controls on Nitrogen Cycling of Biological Soil Crusts,” in *Biological Soil Crusts: An Organizing Principle in Drylands*, eds Weber B. Büdel B. Belnap J. (Cham: Springer International Publishing), 257–285. 10.1007/978-3-319-30214-0_14

[B6] Belnap J. (2002). Nitrogen fixation in biological soil crusts from southeast Utah, USA. *Biol. Fertil. Soils* 35 128–135. 10.1007/s00374-002-0452-x

[B7] Bouskill N. J. Eveillard D. Chien D. Jayakumar A. Ward B. B. (2012). Environmental factors determining ammonia-oxidizing organism distribution and diversity in marine environments. *Environ. Microbiol.* 14 714–729. 10.1111/j.1462-2920.2011.02623.x 22050634

[B8] Bowker M. A. Maestre F. T. Eldridge D. Belnap J. Castillo-Monroy A. Escolar C. (2014). Biological soil crusts (biocrusts) as a model system in community, landscape and ecosystem ecology. *Biodivers. Conserv.* 23 1619–1637. 10.1007/s10531-014-0658-x

[B9] Burgin A. J. Hamilton S. K. (2007). Have we overemphasized the role of denitrification in aquatic ecosystems? A review of nitrate removal pathways. *Front. Ecol. Environ.* 5:89–96. 10.1890/1540-929520075[89:HWOTRO]2.0.CO;2

[B10] Castillo-Monroy A. P. Maestre F. T. Delgado-Baquerizo M. Gallardo A. (2010). Biological soil crusts modulate nitrogen availability in semi-arid ecosystems: insights from a Mediterranean grassland. *Plant Soil* 333 21–34. 10.1007/s11104-009-0276-7

[B11] Castrillo G. Teixeira P. J. Paredes S. H. Law T. F. de Lorenzo L. Feltcher M. E. (2017). Root microbiota drive direct integration of phosphate stress and immunity. *Nature* 543 513–518. 10.1038/nature21417 28297714 PMC5364063

[B12] Cerqueira T. Barroso C. Froufe H. Egas C. Bettencourt R. (2018). Metagenomic Signatures of Microbial Communities in Deep-Sea Hydrothermal Sediments of Azores Vent Fields. *Microb. Ecol.* 76 387–403. 10.1007/s00248-018-1144-x 29354879

[B13] Cole J. A. Brown C. M. (1980). Nitrite Reduction to Ammonia by Fermentative Bacteria: A Short Circuit in the Biological Nitrogen Cycle. *FEMS Microbiol. Lett.* 7 65–72. 10.1111/j.1574-6941.1980.tb01578.x

[B14] Collins S. L. Sinsabaugh R. L. Crenshaw C. Green L. Porras-Alfaro A. Stursova M. (2008). Pulse dynamics and microbial processes in aridland ecosystems. *J. Ecol.* 96 413–420. 10.1111/j.1365-2745.2008.01362.x

[B15] Dahlman L. Persson J. Palmqvist K. Nasholm T. (2004). Organic and inorganic nitrogen uptake in lichens. *Planta* 219 459–467. 10.1007/s00425-004-1247-0 15060826

[B16] Daims H. Lucker S. Wagner M. (2016). A New Perspective on Microbes Formerly Known as Nitrite-Oxidizing Bacteria. *Trends Microbiol.* 24 699–712. 10.1016/j.tim.2016.05.004 27283264 PMC6884419

[B17] Datta M. S. Sliwerska E. Gore J. Polz M. F. Cordero O. X. (2016). Microbial interactions lead to rapid micro-scale successions on model marine particles. *Nat. Commun.* 7:11965. 10.1038/ncomms11965 27311813 PMC4915023

[B18] Delgado-Baquerizo M. Maestre F. T. Escolar C. Gallardo A. Ochoa V. Gozalo B. (2014). Direct and indirect impacts of climate change on microbial and biocrust communities alter the resistance of the N cycle in a semiarid grassland. *J. Ecol.* 102 1592–1605. 10.1111/1365-2745.12303

[B19] Delgado-Baquerizo M. Maestre F. T. Gallardo A. Bowker M. A. Wallenstein M. D. Quero J. L. (2013). Decoupling of soil nutrient cycles as a function of aridity in global drylands. *Nature* 502 672–676. 10.1038/nature12670 24172979

[B20] Ettwig K. F. Butler M. K. Le Paslier D. Pelletier E. Mangenot S. Kuypers M. M. (2010). Nitrite-driven anaerobic methane oxidation by oxygenic bacteria. *Nature* 464 543–548. 10.1038/nature08883 20336137

[B21] Falkowski P. G. Fenchel T. Delong E. F. (2008). The microbial engines that drive Earth’s biogeochemical cycles. *Science* 320 1034–1039. 10.1126/science.1153213 18497287

[B22] Ganesh S. Parris D. J. DeLong E. F. Stewart F. J. (2014). Metagenomic analysis of size-fractionated picoplankton in a marine oxygen minimum zone. *ISME J.* 8 187–211. 10.1038/ismej.2013.144 24030599 PMC3869020

[B23] Geisseler D. Horwath W. R. Joergensen R. G. Ludwig B. (2010). Pathways of nitrogen utilization by soil microorganisms – A review. *Soil Biol. Biochem.* 42 2058–2067. 10.1016/j.soilbio.2010.08.021

[B24] Gravel D. Massol F. Leibold M. A. (2016). Stability and complexity in model meta-ecosystems. *Nat. Commun.* 7:12457. 10.1038/ncomms12457 27555100 PMC4999499

[B25] Grote E. E. Belnap J. Housman D. C. Sparks J. P. (2010). Carbon exchange in biological soil crust communities under differential temperatures and soil water contents: implications for global change. *Glob. Change Biol.* 16 2763–2774. 10.1111/j.1365-2486.2010.02201.x

[B26] Gu C. Riley W. J. (2010). Combined effects of short term rainfall patterns and soil texture on soil nitrogen cycling - a modeling analysis. *J. Contam. Hydrol.* 112 141–154. 10.1016/j.jconhyd.2009.12.003 20116129

[B27] Guo J. Peng Y. Fan L. Zhang L. Ni B. J. Kartal B. (2016). Metagenomic analysis of anammox communities in three different microbial aggregates. *Environ. Microbiol.* 18 2979–2993. 10.1111/1462-2920.13132 26568531

[B28] He Z. L. Gentry T. J. Schadt C. W. Wu L. Y. Liebich J. Chong S. C. (2007). GeoChip: a comprehensive microarray for investigating biogeochemical, ecological and environmental processes. *ISME J.* 1 67–77. 10.1038/ismej.2007.2 18043615

[B29] Hooper D. U. Johnson L. (1999). Nitrogen limitation in dryland ecosystems: Responses to geographical and temporal variation in precipitation. *Biogeochemistry* 46 247–293. 10.1023/A:1006145306009

[B30] Isobe K. Ohte N. (2014). Ecological perspectives on microbes involved in N-cycling. *Microb. Environ.* 29 4–16. 10.1264/jsme2.me13159 24621510 PMC4041230

[B31] Jiang X. Hou X. Zhou X. Xin X. Wright A. Jia Z. (2015). pH regulates key players of nitrification in paddy soils. *Soil Biol. Biochem.* 81 9–16. 10.1016/j.soilbio.2014.10.025

[B32] Johnson S. L. Budinoff C. R. Belnap J. Garcia-Pichel F. (2005). Relevance of ammonium oxidation within biological soil crust communities. *Environ. Microbiol.* 7 1–12. 10.1111/j.1462-2920.2004.00649.x 15643930

[B33] Johnson S. L. Neuer S. Garcia-Pichel F. (2007). Export of nitrogenous compounds due to incomplete cycling within biological soil crusts of arid lands. *Environ. Microbiol.* 9 680–689. 10.1111/j.1462-2920.2006.01187.x 17298368

[B34] Keren R. Lawrence J. E. Zhuang W. Jenkins D. Banfield J. F. Alvarez-Cohen L. (2020). Increased replication of dissimilatory nitrate-reducing bacteria leads to decreased anammox bioreactor performance. *Microbiome* 8:7. 10.1186/s40168-020-0786-3 31980038 PMC6982389

[B35] Keshri J. Yousuf B. Mishra A. Jha B. (2015). The abundance of functional genes, cbbL, nifH, amoA and apsA, and bacterial community structure of intertidal soil from Arabian Sea. *Microbiol. Res.* 175 57–66. 10.1016/j.micres.2015.02.007 25862282

[B36] Kirstein K. Bock E. (1993). Close Genetic-Relationship between Nitrobacter-Hamburgensis Nitrite Oxidoreductase and *Escherichia*-Coli Nitrate Reductases. *Arch. Microbiol.* 160 447–453. 10.1007/Bf00245305 8297210

[B37] Koike I. Hattori A. (1975). Energy Yield of Denitrification: An Estimate from Growth Yield in Continuous Cultures of *Pseudomonas* denitrificans under Nitrate-, Nitrite- and Nitrous Oxide-limited Conditions. *J. Gener. Microbiol.* 88 11–19. 10.1099/00221287-88-1-11 1151328

[B38] Koleff P. Gaston K. J. Lennon J. J. (2003). Measuring beta diversity for presence-absence data. *J. Anim. Ecol.* 72 367–382. 10.1046/j.1365-2656.2003.00710.x

[B39] Kovacs I. A. Palotai R. Szalay M. S. Csermely P. (2010). Community landscapes: an integrative approach to determine overlapping network module hierarchy, identify key nodes and predict network dynamics. *PLoS One* 5:e12528. 10.1371/journal.pone.0012528 20824084 PMC2932713

[B40] Kraft B. Strous M. Tegetmeyer H. E. (2011). Microbial nitrate respiration–genes, enzymes and environmental distribution. *J. Biotechnol.* 155 104–117. 10.1016/j.jbiotec.2010.12.025 21219945

[B41] Kuypers M. M. M. Marchant H. K. Kartal B. (2018). The microbial nitrogen-cycling network. *Nat. Rev. Microbiol.* 16 263–276. 10.1038/nrmicro.2018.9 29398704

[B42] Lan S. B. Ouyang H. L. Wu L. Zhang D. L. Hu C. X. (2017). Biological soil crust community types differ in photosynthetic pigment composition, fluorescence and carbon fixation in Shapotou region of China. *Appl. Soil Ecol.* 111 9–16. 10.1016/j.apsoil.2016.11.009

[B43] Lan S. B. Wu L. Zhang D. L. Hu C. X. (2015). Analysis of environmental factors determining development and succession in biological soil crusts. *Sci. Total Environ.* 538 492–499. 10.1016/j.scitotenv.2015.08.066 26318686

[B44] Lange O. L. Belnap J. Reichenberger H. (1998). Photosynthesis of the cyanobacterial soil−crust lichen Collema tenax from arid lands in southern Utah, USA: role of water content on light and temperature responses of CO2 exchange. *Funct. Ecol.* 12 195–202. 10.1046/j.1365-2435.1998.00192.x

[B45] Li J.-Y. Jin X.-Y. Zhang X.-C. Chen L. Liu J.-L. Zhang H.-M. (2020). Comparative metagenomics of two distinct biological soil crusts in the Tengger Desert, China. *Soil Biol. Biochem.* 140:107637. 10.1016/j.soilbio.2019.107637

[B46] Li R. Q. Li Y. R. Kristiansen K. Wang J. (2008). SOAP: short oligonucleotide alignment program. *Bioinformatics* 24 713–714. 10.1093/bioinformatics/btn025 18227114

[B47] Li W. Z. Godzik A. (2006). Cd-hit: a fast program for clustering and comparing large sets of protein or nucleotide sequences. *Bioinformatics* 22 1658–1659. 10.1093/bioinformatics/btl158 16731699

[B48] Li Y. Jing H. Xia X. Cheung S. Suzuki K. Liu H. (2018). Metagenomic Insights Into the Microbial Community and Nutrient Cycling in the Western Subarctic Pacific Ocean. *Front. Microbiol.* 9:623. 10.3389/fmicb.2018.00623 29670596 PMC5894113

[B49] Limmer C. Drake H. L. (1996). Non-symbiotic N_2_-fixation in acidic and pH-neutral forest soils: aerobic and anaerobic differentials. *Soil Biol. Biochem.* 28 177–183. 10.1016/0038-0717(95)00118-2

[B50] Lin J. T. Stewart V. (1997). Nitrate Assimilation by Bacteria. *Adv. Microb. Physiol.* 39 1–30. 10.1016/s0065-2911(08)60014-4 9328645

[B51] Liu L. Chen H. Liu M. Yang J. R. Xiao P. Wilkinson D. M. (2019). Response of the eukaryotic plankton community to the cyanobacterial biomass cycle over 6 years in two subtropical reservoirs. *ISME J.* 13 2196–2208. 10.1038/s41396-019-0417-9 31053831 PMC6776060

[B52] Liu X. Y. Koba K. Makabe A. Li X. D. Yoh M. Liu C. Q. (2013). Ammonium first: natural mosses prefer atmospheric ammonium but vary utilization of dissolved organic nitrogen depending on habitat and nitrogen deposition. *N. Phytol.* 199 407–419. 10.1111/nph.12284 23692546

[B53] Llorens-Mares T. Yooseph S. Goll J. Hoffman J. Vila-Costa M. Borrego C. M. (2015). Connecting biodiversity and potential functional role in modern euxinic environments by microbial metagenomics. *ISME J.* 9 1648–1661. 10.1038/ismej.2014.254 25575307 PMC4478705

[B54] Lüke C. Speth D. R. Kox M. A. R. Villanueva L. Jetten M. S. M. (2016). Metagenomic analysis of nitrogen and methane cycling in the Arabian Sea oxygen minimum zone. *PeerJ* 4:e1924. 10.7717/peerj.1924 27077014 PMC4830246

[B55] Maier S. Tamm A. Wu D. Caesar J. Grube M. Weber B. (2018). Photoautotrophic organisms control microbial abundance, diversity, and physiology in different types of biological soil crusts. *ISME J.* 12 1032–1046. 10.1038/s41396-018-0062-8 29445133 PMC5864206

[B56] Marusenko Y. Bates S. T. Anderson I. Johnson S. L. Soule T. Garcia-Pichel F. (2013). Ammonia-oxidizing archaea and bacteria are structured by geography in biological soil crustss acrosss North American arid lands. *Ecol. Processes* 2 2–9. 10.1186/2192-1709-2-9

[B57] McCalley C. K. Sparks J. P. (2008). Controls over nitric oxide and ammonia emissions from Mojave Desert soils. *Oecologia* 156 871–881. 10.1007/s00442-008-1031-0 18392857

[B58] McTigue N. D. Gardner W. S. Dunton K. H. Hardison A. K. (2016). Biotic and abiotic controls on co-occurring nitrogen cycling processes in shallow Arctic shelf sediments. *Nat. Commun.* 7:13145. 10.1038/ncomms13145 27782213 PMC5095177

[B59] Moreno-Vivian C. Cabello P. Martinez-Luque M. Blasco R. Castillo F. (1999). Prokaryotic nitrate reduction: Molecular properties and functional distinction among bacterial nitrate reductases. *J. Bacteriol.* 181 6573–6584. 10.1128/JB.181.21.6573-6584.1999 10542156 PMC94119

[B60] Muro-Pastor M. I. Reyes J. C. Florencio F. J. (2005). Ammonium assimilation in cyanobacteria. *Photosynth. Res.* 83 135–150. 10.1007/s11120-004-2082-7 16143848

[B61] Nelson M. B. Berlemont R. Martiny A. C. Martiny J. B. (2015). Nitrogen Cycling Potential of a Grassland Litter Microbial Community. *Appl. Environ. Microbiol.* 81 7012–7022. 10.1128/AEM.02222-15 26231641 PMC4579426

[B62] Nelson M. B. Martiny A. C. Martiny J. B. (2016). Global biogeography of microbial nitrogen-cycling traits in soil. *Proc. Natl. Acad. Sci. U S A.* 113 8033–8040. 10.1073/pnas.1601070113 27432978 PMC4961168

[B63] Noguchi H. Park J. Takagi T. (2006). MetaGene: prokaryotic gene finding from environmental genome shotgun sequences. *Nucleic Acids Res.* 34 5623–5630. 10.1093/nar/gkl723 17028096 PMC1636498

[B64] Olmstead L. B. Alexander L. T. Middleton H. E. (1930). A pipette method of mechanical analysis of soils based on improved dispersion procedure. *Tech. Bull. U S. Depart. Agricult.* 1930 1–23. 10.22004/ag.econ.158882

[B65] Ouyang H. L. Hu C. X. (2017). Insight into climate change from the carbon exchange of biocrusts utilizing non-rainfall water. *Sci. Rep.* 7:2573. 10.1038/s41598-017-02812-y 28566698 PMC5451392

[B66] Ouyang H. L. Lan S. B. Yang H. J. Hu C. X. (2017). Mechanism of biocrusts boosting and utilizing non-rainfall water in Hobq Desert of China. *Appl. Soil Ecol.* 120 70–80. 10.1016/j.apsoil.2017.07.024

[B67] Peng Y. Leung H. C. Yiu S. M. Chin F. Y. (2012). IDBA-UD: a de novo assembler for single-cell and metagenomic sequencing data with highly uneven depth. *Bioinformatics* 28 1420–1428. 10.1093/bioinformatics/bts174 22495754

[B68] Pepe-Ranney C. Koechli C. Potrafka R. Andam C. Eggleston E. Garcia-Pichel F. (2016). Non-cyanobacterial diazotrophs mediate dinitrogen fixation in biological soil crusts during early crust formation. *ISME J.* 10 287–298. 10.1038/ismej.2015.106 26114889 PMC4737922

[B69] Pett-Ridge J. Silver W. L. Firestone M. K. (2006). Redox Fluctuations Frame Microbial Community Impacts on N-cycling Rates in a Humid Tropical Forest Soil. *Biogeochemistry* 81 95–110. 10.1007/s10533-006-9032-8

[B70] Qin J. J. Li Y. R. Cai Z. M. Li S. H. Zhu J. F. Zhang F. (2012). A metagenome-wide association study of gut microbiota in type 2 diabetes. *Nature* 490 55–60. 10.1038/nature11450 23023125

[B71] Rasigraf O. Schmitt J. Jetten M. S. M. Luke C. (2017). Metagenomic potential for and diversity of N-cycle driving microorganisms in the Bothnian Sea sediment. *Microbiologyopen* 6:e475. 10.1002/mbo3.475 28544522 PMC5552932

[B72] Ren M. Zhang Z. Wang X. Zhou Z. Chen D. Zeng H. (2018). Diversity and Contributions to Nitrogen Cycling and Carbon Fixation of Soil Salinity Shaped Microbial Communities in Tarim Basin. *Front. Microbiol.* 9:431. 10.3389/fmicb.2018.00431 29593680 PMC5855357

[B73] Reverey F. Grossart H.-P. Premke K. Lischeid G. (2016). Carbon and nutrient cycling in kettle hole sediments depending on hydrological dynamics: a review. *Hydrobiologia* 775 1–20. 10.1007/s10750-016-2715-9

[B74] Rosch C. Mergel A. Bothe H. (2002). Biodiversity of Denitrifying and Dinitrogen-Fixing Bacteria in an Acid Forest Soil. *Appl. Environ. Microbiol.* 68 3818–3829. 10.1128/aem.68.8.3818-3829.2002 12147477 PMC124007

[B75] Rütting T. Boeckx P. Müller C. Klemedtsson L. (2011). Assessment of the importance of dissimilatory nitrate reduction to ammonium for the terrestrial nitrogen cycle. *Biogeosciences* 8 1779–1791. 10.5194/bg-8-1779-2011

[B76] Saarenheimo J. Tiirola M. A. Rissanen A. J. (2015). Functional gene pyrosequencing reveals core proteobacterial denitrifiers in boreal lakes. *Front. Microbiol.* 6:674. 10.3389/fmicb.2015.00674 26191058 PMC4486872

[B77] Schulz S. Brankatschk R. Dümig A. Kögel-Knabner I. Schloter M. Zeyer J. (2013). The role of microorganisms at different stages of ecosystem development for soil formation. *Biogeosciences* 10 3983–3996. 10.5194/bg-10-3983-2013

[B78] Seitz H.-J. Cypionka H. (1986). Chemolithotrophic growth of Desulfovibrio desulfuricans with hydrogen coupled to ammonification of nitrate or nitrite. *Arch. Microbiol.* 146 63–67. 10.1007/BF00690160

[B79] Sgouridis F. Heppell C. M. Wharton G. Lansdown K. Trimmer M. (2011). Denitrification and dissimilatory nitrate reduction to ammonium (DNRA) in a temperate re-connected floodplain. *Water Res.* 45 4909–4922. 10.1016/j.watres.2011.06.037 21813153

[B80] Shannon P. Markiel A. Ozier O. Baliga N. S. Wang J. T. Ramage D. (2003). Cytoscape: A software environment for integrated models of biomolecular interaction networks. *Genome Res.* 13 2498–2504. 10.1101/gr.1239303 14597658 PMC403769

[B81] Simon J. Klotz M. G. (2013). Diversity and evolution of bioenergetic systems involved in microbial nitrogen compound transformations. *Biochim. Biophys. Acta* 1827 114–135. 10.1016/j.bbabio.2012.07.005 22842521

[B82] Šmilauer P. Lepš J. (2014). *Multivariate analysis of ecological data using CANOCO 5.* New York: Cambridge University Press.

[B83] Souza R. C. Hungria M. Cantão M. E. Vasconcelos A. T. R. Nogueira M. A. Vicente V. A. (2015). Metagenomic analysis reveals microbial functional redundancies and specificities in a soil under different tillage and crop-management regimes. *Appl. Soil Ecol.* 86 106–112. 10.1016/j.apsoil.2014.10.010

[B84] Stein L. Y. Klotz M. G. (2016). The nitrogen cycle. *Curr. Biol.* 26 R94–R98. 10.1016/j.cub.2015.12.021 26859274

[B85] Strauss S. L. Day T. A. Garcia-Pichel F. (2011). Nitrogen cycling in desert biological soil crusts across biogeographic regions in the Southwestern United States. *Biogeochemistry* 108 171–182. 10.1007/s10533-011-9587-x

[B86] Thamdrup B. (2012). New Pathways and Processes in the Global Nitrogen Cycle. *Annu. Rev. Ecol. Evolut. Systemat.* 43 407–428. 10.1146/annurev-ecolsys-102710-145048

[B87] Throback I. N. Enwall K. Jarvis A. Hallin S. (2004). Reassessing PCR primers targeting nirS, nirK and nosZ genes for community surveys of denitrifying bacteria with DGGE. *FEMS Microb. Ecol.* 49 401–417. 10.1016/j.femsec.2004.04.011 19712290

[B88] Tiedje J. M. (1988). “Ecology of denitrification and dissimilatory nitrate reduction to ammonium,” in *Environmental Microbiology of Anaerobes*, ed. Zehnder A. J. B. (New York, N.Y.: John Wiley and Sons), 179–244.

[B89] Tiedje J. M. Sexstone A. J. Myrold D. D. Robinson J. A. (1982). Denitrification: ecological niche, competition and survival. *Antonie van Leeuwenhoek* 48 569–583. 10.1007/BF00399542 6762848

[B90] Tu Q. He Z. Wu L. Xue K. Xie G. Chain P. (2017). Metagenomic reconstruction of nitrogen cycling pathways in a CO_2_-enriched grassland ecosystem. *Soil Biol. Biochem.* 106 99–108. 10.1016/j.soilbio.2016.12.017

[B91] Vance E. D. Brookes P. C. Jenkinson D. S. (1987). An extraction method for measuring soil microbial biomass-C. *Soil Biol. Biochem.* 19 703–707. 10.1016/0038-0717(87)90052-6

[B92] Varin T. Lovejoy C. Jungblut A. D. Vincent W. F. Corbeil J. (2010). Metagenomic profiling of Arctic microbial mat communities as nutrient scavenging and recycling systems. *Limnol. Oceanogr.* 55 1901–1911. 10.4319/lo.2010.55.5.1901

[B93] Wang J. Bao J. T. Li X. R. Liu Y. B. (2016). Molecular Ecology of nifH Genes and Transcripts Along a Chronosequence in Revegetated Areas of the Tengger Desert. *Microb. Ecol.* 71 150–163. 10.1007/s00248-015-0657-9 26276410

[B94] Wang M. Veldsink J. H. Dini-Andreote F. Salles J. F. (2018a). Compositional and abundance changes of nitrogen-cycling genes in plant-root microbiomes along a salt marsh chronosequence. *Antonie Van Leeuwenhoek* 111 2061–2078. 10.1007/s10482-018-1098-5 29846874

[B95] Wang X. Hu M. Ren H. Li J. Tong C. Musenze R. S. (2018b). Seasonal variations of nitrous oxide fluxes and soil denitrification rates in subtropical freshwater and brackish tidal marshes of the Min River estuary. *Sci. Total Environ.* 616-617 1404–1413. 10.1016/j.scitotenv.2017.10.175 29122343

[B96] Wang S. Zhu G. Zhuang L. Li Y. Liu L. Lavik G. (2020). Anaerobic ammonium oxidation is a major N-sink in aquifer systems around the world. *ISME J.* 14 151–163. 10.1038/s41396-019-0513-x 31595050 PMC6908648

[B97] Wang Y. Li C. Kou Y. Wang J. Tu B. Li H. (2017). Soil pH is a major driver of soil diazotrophic community assembly in Qinghai-Tibet alpine meadows. *Soil Biol. Biochem.* 115 547–555. 10.1016/j.soilbio.2017.09.024

[B98] Weber B. Wu D. Tamm A. Ruckteschler N. Rodriguez-Caballero E. Steinkamp J. (2015). Biological soil crusts accelerate the nitrogen cycle through large NO and HONO emissions in drylands. *Proc. Natl. Acad. Sci. U S A.* 112 15384–15389. 10.1073/pnas.1515818112 26621714 PMC4687600

[B99] Yang Y. F. Wu L. W. Lin Q. Y. Yuan M. T. Xu D. P. Yu H. (2013). Responses of the functional structure of soil microbial community to livestock grazing in the Tibetan alpine grassland. *Glob. Change Biol.* 19 637–648. 10.1111/gcb.12065 23504798

[B100] Yeager C. M. Kuske C. R. Carney T. D. Johnson S. L. Ticknor L. O. Belnap J. (2012). Response of biological soil crust diazotrophs to season, altered summer precipitation, and year-round increased temperature in an arid grassland of the colorado plateau, USA. *Front. Microbiol.* 3:358. 10.3389/fmicb.2012.00358 23087679 PMC3468842

[B101] Zaady E. (2005). Seasonal Change and Nitrogen Cycling in a Patchy Negev Desert: a Review. *Arid Land Res. Manage.* 19 111–124. 10.1080/15324980590916512

[B102] Zehr J. P. Capone D. G. (2020). Changing perspectives in marine nitrogen fixation. *Science* 368:eaay9514. 10.1126/science.aay9514 32409447

[B103] Zhang Q. Y. Wang Q. Ouyang H. L. Lan S. B. Hu C. X. (2018). Pyrosequencing reveals significant changes in microbial communities along the ecological succession of biological soil crusts in the Tengger Desert of China. *Pedosphere* 28 350–362. 10.1016/s1002-0160(17)60477-6

[B104] Zheng M. Chen H. Li D. Luo Y. Mo J. (2020). Substrate stoichiometry determines nitrogen fixation throughout succession in southern Chinese forests. *Ecol. Lett.* 23 336–347. 10.1111/ele.13437 31802606

[B105] Zheng Z. J. Zhong W. D. Liu L. Wu C. Y. Zhang L. S. Cai S. F. (2016). Bioinformatics approaches for human gut microbiome research. *Infect. Dis. Translat. Med.* 2 69–79. 10.11979/idtm.201602005

[B106] Zhou X. Tao Y. Yin B. Tucker C. Zhang Y. (2020). Nitrogen pools in soil covered by biological soil crusts of different successional stages in a temperate desert in Central Asia. *Geoderma* 366:114166. 10.1016/j.geoderma.2019.114166

