# Supplementary material for: Metagenomic Insight Into Patterns and Mechanism of Nitrogen Cycle During Biocrust Succession
Source: Front Microbiol. 2021 Mar 17;12:633428. doi: 10.3389/fmicb.2021.633428 (PMC8009985; doi:10.3389/fmicb.2021.633428)
Supplement: Supplementary Figure 1 — Schematic of the experimental and analytical workflow. A, C, G, M represent four types of natural biocrusts at different succession stages. A: algae crusts, C: cyanolichen crusts, G: chlorolichen crusts, M: moss crusts. Ae, Am, and Al represent early, middle, and later stage of simulated cyanobacterial crusts, respectively. [file Data_Sheet_1.docx]

**Supplementary Tables**

**Supplementary Table S1** The qPCR primers and conditions used in this paper.

| Primers | Sequence (5' -3') | Annealing temperature and time | Efficiency (%) | Source of calibration standard | Reference |
| --- | --- | --- | --- | --- | --- |
| **Taxon/Group specific qPCR** | | | | | |
| **Bacteria** | | | | | |
| Eub338 | ACT CCT ACG GGA GGC AGC AG | 53 °C, 15 s | 95.79 | *Alsobacter* sp.  strain SH9  (MG654667; 100%) | Fierer et al., 2005 |
| Eub518 | ATT ACC GCG GCT GCT GG |  |  |  |  |
| **Fungi** | | | | | |
| NL1f | ATATCAATAAGCGGAGGAAAAG | 60 °C, 15 s | 89.35 | *Arachnomyces* sp. BDS-2017a  (KY440754; 98.79%) | Bates et al., 2010 |
| LS2r | ATTCCCAAACAACTCGACTC |  |  |  |  |
| **Archaea** | | | | | |
| arch967F | AATTGGCGGGGGAGCAC | 58 °C, 15 s | 96.73 | *Candidatus* *Nitrososphaera* *gargensis* (CP002408; 99.27%) | Cadillo-Quiroz et al., 2006 |
| arch1060R | GGCCATGCACCWCCTCTC |  |  |  |  |
| **Cyanobacteria** | | | | | |
| CYA359F | GGGGAATYTTCCGCAATGGG | 60 °C, 30 s | 83.85 | *Scytonema* sp. WJT9-NPBG6B (KF934172.1; 100%) | Nubel et al., 1997 |
| CYA781R_a | GACTACTGGGGTATCTAATCCCATT |  |  |  |  |
| CYA781R_b | GACTACAGGGGTATCTAATCCCTTT |  |  |  |  |
| **qPCR of genes involved in nitrogen cycling** | | | | | |
| ***nifH*** | | | | | |
| nifH-F | AAAGGYGGWATCGGYAARTCCACCAC | 55 °C, 30 s | 95.76 | Scytonema sp. FGP-7A  (DQ531669; 99%) | Rosch et al., 2002 |
| nifH-R | TTGTTSGCSGCRTACATSGCCATCAT |  |  |  |  |
| ***nrfA*** |  |  |  |  |  |
| nrfA_F2aw | CARTGYCAYGTBGARTA | 58 °C, 30 s | 66.12 | Uncultured bacterium clone HCB9 (JX293778.1; 74.04%) | Wu et al., 2019 |
| nrfA_R1 | TWNGGCATRTGRCARTC |  |  |  |  |

**Supplementary Table S2** Assembly and annotation statistics of metagenome and 16S rRNA gene sequence. (ORF, open reading frame; QC, quality control)

|  | **Natural biocrusts** | **Simulated biocrusts** |
| --- | --- | --- |
| **Metagenome sequencing** |  |  |
| Platform | Illumina HiSeq 2000 (101-bp paired ends) for biocrusts in 2015;  Illumina Xten (150-bp paired ends) for biocrusts in 2016, 2017, and the first batch in 2018  Illumina NovaSeq (150-bp paired ends) for biocrusts of the second batch in 2018 | Illumina NovaSeq  (150-bp paired ends) |
| Raw reads | 2 612 994 856 paired-end reads (2015, 2016, 2017)  3 159 666 872 (batch 1 in 2018, the same below) | 1 324 855 240 paired-end reads |
|  | 3 264 404 632 (batch 2 in 2018, the same below) |  |
| Reads post QC | 2 425 542 258 (avg. length 127 bp)  3 050 673 626 (avg. length 150 bp) | 1 308 379 205  (avg. length 150 bp) |
|  | 3 229 586 996 (avg. length 150 bp) |  |
| Contigs | 28 869 443  58 927 535 | 17 188 980 |
|  | 60 229 473 |  |
| N50 of contigs | 914 bp  1037 bp | 1 275 bp |
|  | 941 bp |  |
| Longest contig | 1 216 310 bp  515 942 bp | 375 362 bp |
|  | 240 939 bp |  |
| Predicted ORFs | 40 754 956  98 019 551 | 28 487 007 |
|  | 94 909 751 |  |
| ORFs after 95% clustering | 31 201 753 (avg. length 490 bp)  56 563 945 (avg. length 483 bp) | 17 460 047 (avg. length 527 bp) |
|  | 56 345 535 (avg. length 488 bp) |  |
| Percent of ORFs annotated | avg. 91% in NR; 40% in KEGG | avg. 90% in NR; 42% in KEGG |
| **16S rRNA gene sequencing** | | |
| Raw sequence number | 2 942 905 (all batches analyzed together) | 450 202 |
| Valid sequence number | 1 893 138 | 372 379 |
| OTUs | 3 525 | 1 892 |
| coverage | 97.43% | 98.08% |

**Supplementary Table S3** The corresponding relationship of N-cycle pathways, genes and KEGG Orthology (KO) number.

| pathway-1 | pathway-2 | gene | KO | Definition |
| --- | --- | --- | --- | --- |
| Nitrogen fixation | Nitrogen fixation | *nifDHK* | K02586 | nifD; nitrogenase molybdenum-iron protein alpha chain [EC:1.18.6.1] |
|  |  |  | K02588 | nifH; nitrogenase iron protein NifH [EC:1.18.6.1] |
|  |  |  | K02591 | nifK; nitrogenase molybdenum-iron protein beta chain [EC:1.18.6.1] |
| Nitrification | Ammonia oxidation | *amoABC* | K10944 | pmoA-amoA; methane/ammonia monooxygenase subunit A [EC:1.14.18.3 1.14.99.39] |
|  |  |  | K10945 | pmoB-amoB；methane/ammonia monooxygenase subunit B |
|  |  |  | K10946 | pmoC-amoC; methane/ammonia monooxygenase subunit C |
|  | hydroxylamine oxidation | *hao* | K10535 | hao;hydroxylamine dehydrogenase [EC:1.7.2.6] |
|  | Nitrite oxidation | *nxrAB* | K00370 | narG, narZ, nxrA; nitrate reductase / nitrite oxidoreductase, alpha subunit [EC:1.7.5.1 1.7.99.4] |
|  |  |  | K00371 | narH, narY, nxrB; nitrate reductase / nitrite oxidoreductase, beta subunit [EC:1.7.5.1 1.7.99.4] |
| Nitrate reduction | DNRN | *narGHI* | K00370 | narG, narZ, nxrA; nitrate reductase / nitrite oxidoreductase, alpha subunit [EC:1.7.5.1 1.7.99.4] |
|  |  |  | K00371 | narH, narY, nxrB; nitrate reductase / nitrite oxidoreductase, beta subunit [EC:1.7.5.1 1.7.99.4] |
|  |  |  | K00374 | narI, narV; nitrate reductase gamma subunit [EC:1.7.5.1 1.7.99.4] |
|  |  | *napAB* | K02567 | napA; periplasmic nitrate reductase NapA [EC:1.7.99.4] |
|  |  |  | K02568 | napB; cytochrome c-type protein NapB |
| Denitrification | Denitrification | *nirK* | K00368 | nirK; nitrite reductase (NO-forming) [EC:1.7.2.1] |
|  |  | *nirS* | K15864 | nirS; nitrite reductase (NO-forming) / hydroxylamine reductase [EC:1.7.2.1 1.7.99.1] |
|  |  | *norBC* | K04561 | norB; nitric oxide reductase subunit B [EC:1.7.2.5] |
|  |  |  | K02305 | norC; nitric oxide reductase subunit C |
|  |  | *nosZ* | K00376 | nosZ; nitrous-oxide reductase [EC:1.7.2.4] |
| DNiRA | DNiRA | *nirBD* | K00362 | nirB; nitrite reductase (NADH) large subunit [EC:1.7.1.15] |
|  |  |  | K00363 | nirD; nitrite reductase (NADH) small subunit [EC:1.7.1.15] |
|  |  | *nrfAH* | K03385 | nrfA; nitrite reductase (cytochrome c-552) [EC:1.7.2.2] |
|  |  |  | K15876 | nrfH; cytochrome c nitrite reductase small subunit |
| ANRA | ANRN | *nasAB* | K00372 | nasA; assimilatory nitrate reductase catalytic subunit [EC:1.7.99.4] |
|  |  |  | K00360 | nasB; assimilatory nitrate reductase electron transfer subunit [EC:1.7.99.4] |
|  |  | *narB* | K00367 | narB; ferredoxin-nitrate reductase [EC:1.7.7.2] |
|  |  | *NR* | K10534 | NR; nitrate reductase (NAD(P)H) [EC:1.7.1.1 1.7.1.2 1.7.1.3] |
|  | ANiRA | *nirA* | K00366 | nirA; ferredoxin-nitrite reductase [EC:1.7.7.1] |
|  |  | *NIT-6* | K17877 | NIT-6; nitrite reductase (NAD(P)H) [EC:1.7.1.4] |
| hcp | hcp | *hcp* | K05601 | hcp; hydroxylamine reductase [EC:1.7.99.1] |
| Anammox | *hzsABC* | *hzsA* | K20932 | hydrazine synthase subunit A |
|  |  | *hzsB* | K20933 | hydrazine synthase subunit B |
|  |  | *hzsC* | K20934 | hydrazine synthase subunit C |
|  | *hzo* | *hzo* | K20935 | hydrazine dehydrogenase |
| Ammonium assimilation | GS/GOGAT | *glnA* | K01915 | glnA, GLUL; glutamine synthetase [EC:6.3.1.2] |
|  |  | *glt* | K00264 | GLT1; glutamate synthase (NADPH/NADH) [EC:1.4.1.13 1.4.1.14] |
|  |  |  | K00265 | gltB; glutamate synthase (NADPH/NADH) large chain [EC:1.4.1.13 1.4.1.14] |
|  |  |  | K00266 | gltD; glutamate synthase (NADPH/NADH) small chain [EC:1.4.1.13 1.4.1.14] |
|  |  |  | K00284 | E1.4.7.1; glutamate synthase (ferredoxin) [EC:1.4.7.1] |
|  | gdh | *gdh* | K00260 | gudB, rocG; glutamate dehydrogenase [EC:1.4.1.2] |
|  |  |  | K15371 | GDH2; glutamate dehydrogenase [EC:1.4.1.2] |
|  |  |  | K00261 | GLUD1_2, gdhA; glutamate dehydrogenase (NAD(P)+) [EC:1.4.1.3] |
|  |  |  | K00262 | E1.4.1.4, gdhA; glutamate dehydrogenase (NADP+) [EC:1.4.1.4] |
| Mineralization | urease | *ure* | K01427 | URE; urease [EC:3.5.1.5] |
|  |  |  | K01428 | ureC; urease subunit alpha [EC:3.5.1.5] |
|  |  |  | K01429 | ureB; urease subunit beta [EC:3.5.1.5] |
|  |  |  | K01430 | ureA; urease subunit gamma [EC:3.5.1.5] |
|  |  |  | K14048 | ureAB; urease subunit gamma/beta [EC:3.5.1.5] |
|  | glutaminase | *gls* | K01425 | glsA, GLS; glutaminase [EC:3.5.1.2] |

**Supplementary Table S4** The properties of biocrusts. Data are reported as average ±SD. Values with different letters are significantly different at 0.05 level within natural biocrusts (n=3) or simulated biocrusts (n=4). WC: water content; EC, electrical conductivity; Eh, redox potential; TC, total carbon; TOC, total organic carbon; DOC, dissolved organic carbon; BC: biomass carbon; RC: remnant carbon; TN, total nitrogen; TP, total phosphorus; DON, dissolved organic nitrogen. ND: not detected; NC: not collected.

|  | 15A | 15C | 15G | 15M | Ae | Am | Al |
| --- | --- | --- | --- | --- | --- | --- | --- |
| DNA (μg g^-1^ soil) | 6.39±0.25^c^ | 17.37±0.67^a^ | 17.57±2.36^a^ | 12.67±0.95^b^ | 0.35±0.17^b^ | 0.83±0.19^a^ | 0.93±0.18^a^ |
| Bacteria (🞨10^9^ copies g^-1^ soil) | 5.58±2.69^a^ | 9.39±1.15^a^ | 6.66±1.34^a^ | 8.11±2.81^a^ | 0.19±0.06^b^ | 0.9±0.17^a^ | 1.06±0.23^a^ |
| Cyanobacteria (🞨10^8^ copies g^-1^ soil) | 26.53±3.65^a^ | 17.75±4.95^b^ | 9.80 ±2.13^c^ | 5.59 ±1.16^c^ | 0.09±0.03^b^ | 3.99±0.81^a^ | 5.07±0.91^a^ |
| Fungi (🞨10^8^ copies g^-1^ soil) | 0.33±0.09^b^ | 3.14±0.01^b^ | 32.13±9.62^a^ | 1.88±0.51^b^ | 0.09±0.03^c^ | 0.7±0.13^b^ | 0.94±0.11^a^ |
| Archaea (🞨10^8^ copies g^-1^ soil) | 0.94±0.26^a^ | 0.98±0.52^a^ | 0.89±0.5^a^ | 1.11±0.91^a^ | 0.15±0.03^a^ | 0.2±0.04^a^ | 0.17±0.04^a^ |
| WC (%) | 0.52±0.08^b^ | 0.70±0.02^a^ | 0.68±0.09^a^ | 0.78±0.04^a^ | 1.85±0.75^a^ | 1.39±0.21^a^ | 1.06±0.49^a^ |
| pH | 7.85±0.05^a^ | 7.80±0.10^ab^ | 7.71±0.07^ab^ | 7.68±0.10^b^ | 9.37±0.02^c^ | 9.54±0.04^a^ | 9.45±0.03^b^ |
| EC (μs·cm^-1^) | 142.15±2.85^d^ | 215.80±3.51^c^ | 444.95±12.11^a^ | 262.53±8.94^b^ | 147.50±5.00^b^ | 220.00±11.55^a^ | 232.50±5.00^a^ |
| Eh (mV) | 348.53±2.29^d^ | 379.84±4.02^c^ | 396.71±5.03^b^ | 411.51±1.42^a^ | 287.8±19.74^b^ | 317.03±7.17^a^ | 323.78±3.86^a^ |
| TC (×10 g kg^-1^) | 69.11±2.10^d^ | 391.32±8.01^b^ | 326.71±7.41^c^ | 474.72±27.21^a^ | 4.99±0.90^a^ | 5.09±0.23^a^ | 4.88±0.64^a^ |
| TOC (g kg^-1^) | 418.67±16.04^d^ | 2520.67±38.55^b^ | 2196±9.85^c^ | 3446.33±84.11^a^ | 0.96±0.04^a^ | 0.55±0.03^c^ | 0.65±0.04^b^ |
| DOC (mg kg^-1^) | 287.61±5.28^d^ | 969.76±28.48^c^ | 1444.42±6.59^b^ | 2197.13±218.89^a^ | 30.59±4.32^b^ | 31.1±2.01^b^ | 41.02±0.89^a^ |
| BC (mg kg^-1^) | 1484.55±18.71^a^ | 1654.73±112.03^a^ | 1304.08±178.98^a^ | 790.46±409.55^b^ | 15.12±6.3^c^ | 51.07±8.14^a^ | 27.33±4.97^b^ |
| RC (g kg^-1^) | 417.18±16.04^d^ | 2519.01±38.63^c^ | 2194.7±9.97^b^ | 3445.54±84.45^a^ | 0.95±0.04^a^ | 0.49±0.03^c^ | 0.62±0.04^b^ |
| CO_3_^2-^(mg kg^-1^) | ND | ND | ND | ND | 89.76±11.55^a^ | 84.76±9.97^a^ | 64.86±10.02^b^ |
| HCO_3_^-^(mg kg^-1^) | 347.27±17.81^c^ | 621.88±31.19^b^ | 813.71±32.38^a^ | 663.51±17.99^b^ | 410.64±19.16^ab^ | 440.99±34.73^a^ | 395.61±11.74^b^ |
| NH_4_^+^ (mg kg^-1^) | 17.48±0.42^b^ | 21.42±1.08^a^ | 18.26±2.72^ab^ | 13.98±1.98^c^ | 9.22±0.32^a^ | 5.12±0.19^b^ | 5.41±0.63^b^ |
| NO_3_^-^ (mg kg^-1^) | 13.05±0.54^b^ | 10.69±0.35^b^ | 7.74±0.20^b^ | 26.62±5.62^a^ | 11.80±0.20^a^ | 2.02±0.06^c^ | 4.67±0.32^b^ |
| NO_2_^-^ (×10^-1^ mg kg^-1^) | 41.01±0.5^ab^ | 19.48±1.41^b^ | 24.91±4.8^ab^ | 51.56±27.94^a^ | 8.52±0.04^a^ | 8.34±0.05^a^ | 8.56±0.22^a^ |
| TN (mg kg^-1^) | 282.39±64.58^b^ | 1135.39±69.60^a^ | 1256.32±161.33^a^ | 1120.91±172.33^a^ | 87.8±3.99^a^ | 90.52±20.52^a^ | 84.89±10.68^a^ |
| DON (mg kg^-1^) | 247.75±64.57^b^ | 1101.34±69.15^a^ | 1227.83±163.17^a^ | 1075.15±172.09^a^ | 65.93±4.16^a^ | 82.55±20.53^a^ | 73.95±10.43^a^ |
| TP (mg kg^-1^) | 92.84±2.63^d^ | 366.02±15.6^a^ | 263.96±10.38^b^ | 197.23±22.75^c^ | 66.38±2.89^a^ | 56.63±6.67^b^ | 55.53±3.20^b^ |
| PO_4_^3-^ (mg kg^-1^) | 4.54±0.08^b^ | 6.94±0.34^a^ | 4.09±0.81^b^ | 4.42±1.44^b^ | 4.11±0.07^a^ | 3.55±0.06^b^ | 3.25±0.14^c^ |
| SO_4_^2-^ (mg kg^-1^) | 84.67±3.85^d^ | 105.64±1.82^b^ | 634.04±5.49^a^ | 92.24±2.35^c^ | 51.94±27.4^b^ | 85.24±20.33^a^ | 94.36±1.03^a^ |
| Ca^2+^ (mg kg^-1^) | 224.16±1.68c | 138.86±4.62d | 556.99±10.3a | 248.1±4.14b | 206.7±8.11^b^ | 222.59±11.23^a^ | 231.48±10.06^a^ |
| Mg^2+^ (mg kg^-1^) | 14.31±0.16c | 10.95±0.37d | 31.04±0.68a | 24.44±0.66b | 30.15±1.21^c^ | 39.99±1.41^b^ | 46.51±1.51^a^ |
| sand (%) | 85.05±2.1^a^ | 63.90±2.77^c^ | 69.00±1.19^b^ | 57.16±2.64^d^ | 99.50±0.25^a^ | 98.32±0.85^a^ | 97.85±1.56^a^ |
| silt (%) | 14.22±2.04^d^ | 34.45±3.02^b^ | 29.69±1.3^c^ | 41.83±2.66^a^ | 0.33±0.29^a^ | 0.35±0.86^a^ | 0.49±0.97^a^ |
| clay (%) | 0.73±0.07^b^ | 1.65±0.52^a^ | 1.32±0.26^a^ | 1.01±0.27^b^ | 0.17±0.04^a^ | 1.33±0.95^a^ | 1.36±0.89^a^ |

**Supplementary figures**


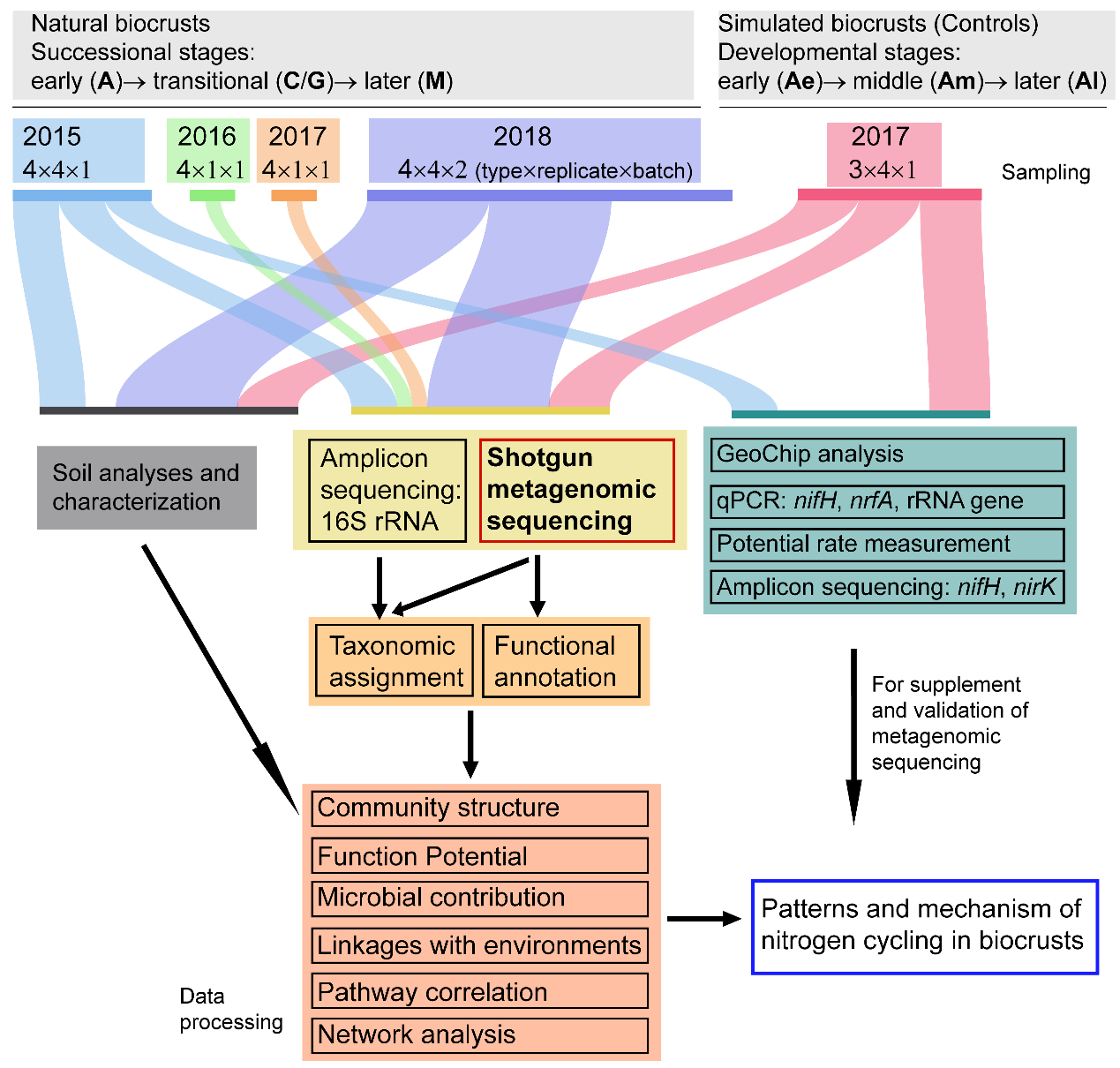


**Supplementary Figure S1** Schematic of the experimental and analytical workflow. A, C, G, M represent four types of natural biocrusts at different succession stages. A: algae crusts, C: cyanolichen crusts, G: chlorolichen crusts, M: moss crusts. Ae, Am, and Al represent early, middle, and later stage of simulated cyanobacterial crusts, respectively.


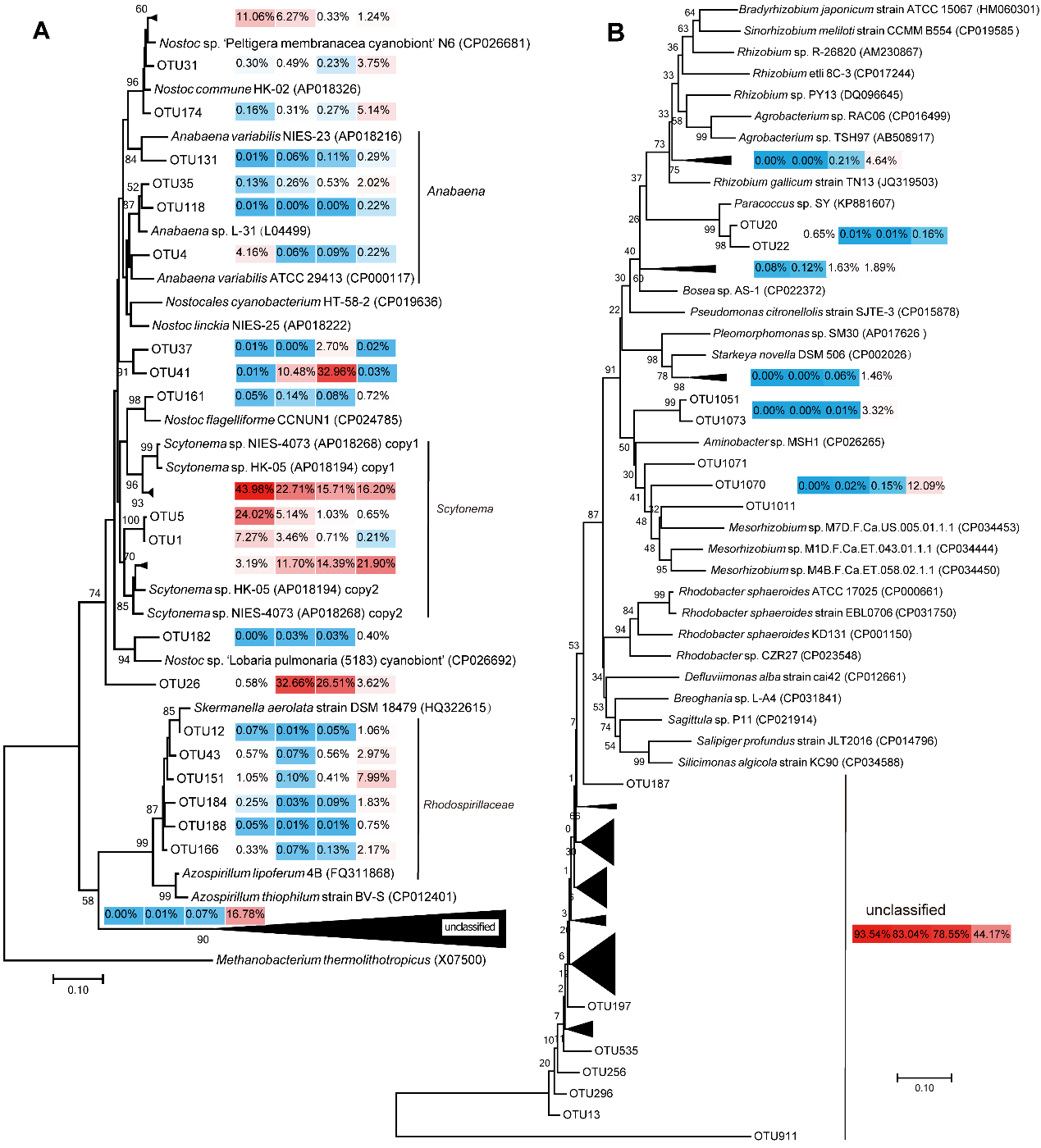


**Supplementary Figure S2** Phylogeny of *nifH* (**A**) and *nirK* (**B**) genes for natural biocrusts in 2015. The heatmap and percentage from left to right are responding to the four types of natural biocrusts: algae crusts, cyanolichen crusts, chlorolichen crusts, and moss crusts, respectively. The evolutionary history was inferred using the Neighbor-Joining method in MEGA7.


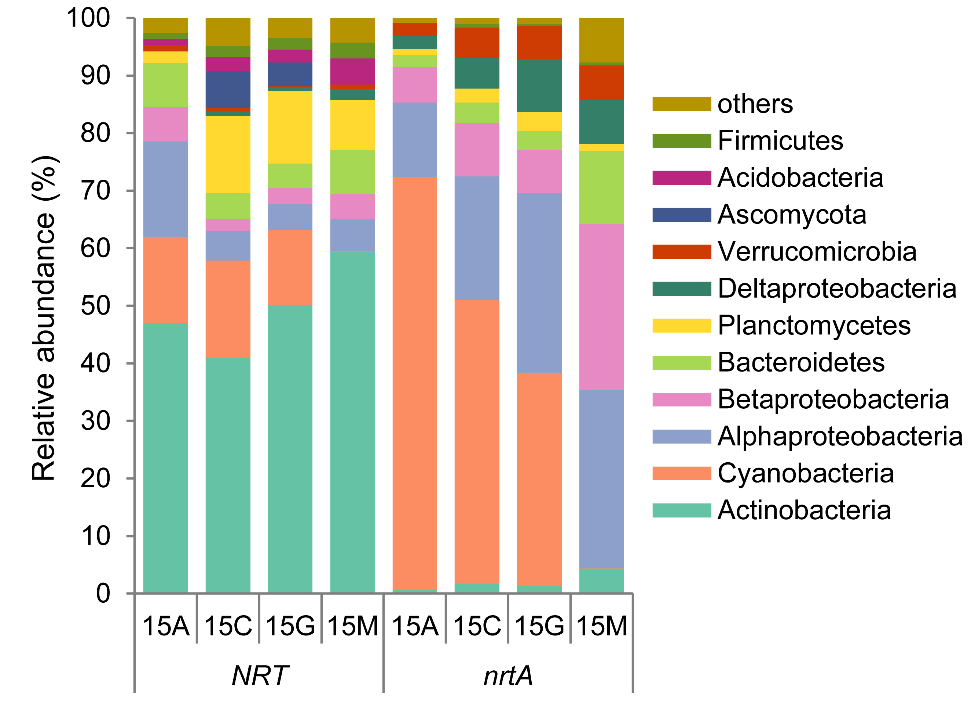


**Supplementary Figure S3** The potential microbial community involved in the transport of nitrate and nitrite in natural biocrusts in 2015. *NRT*, corresponding to K02575, encoding nitrate/nitrite transporter; *nrtA*, corresponding to K15576, encoding nitrate/nitrite transport system substrate-binding protein. A: algae crusts, C: cyanolichen crusts, G: chlorolichen crusts, M: moss crusts.


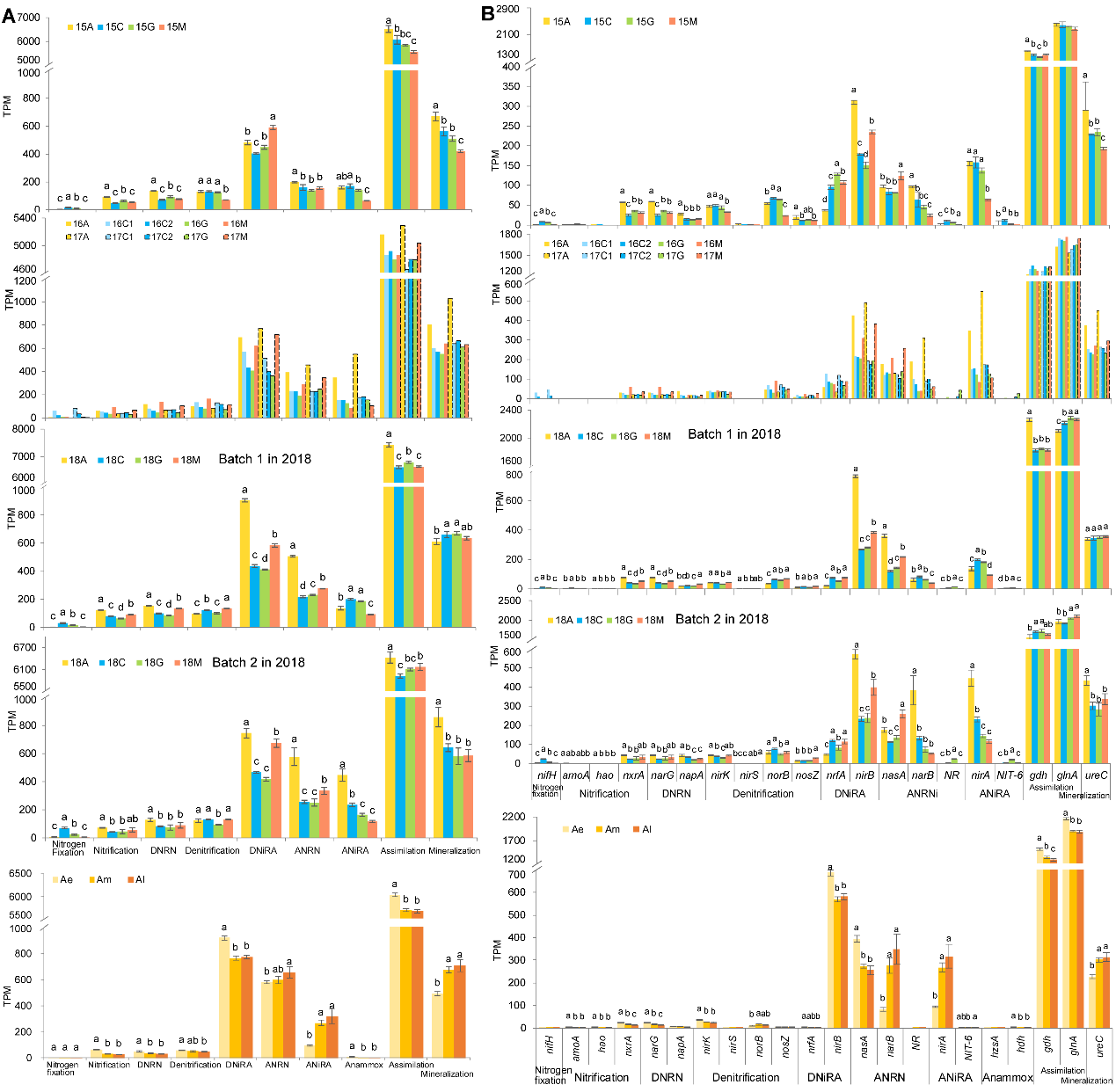


**Supplementary Figure S4** The relative abundance of N-cycle pathways (**A**) or genes (**B**) based on metagenomic analysis in natural biocrusts (5 batches) and simulated biocrusts.

**Supplementary Materials and Methods**

**Bioinformatics for Illumina MiSeq sequencing data**

The sequencing data of 16S rRNA gene were analyzed on the free online platform of Majorbio Cloud Platform (www.majorbio.com). Low-quality raw reads were filtered using fastp (https://github.com/OpenGene/fastp) with following criteria : (1) reads were truncated at any site with an average quality score < 20 over a 50 bp sliding window, and reads shorter than 50 bp after truncated, reads containing ambiguous characters were removed; (2) 1 or more mismatch in barcode; (3) >2 nucleotide mismatch in primers. Paired-end reads were merged using FLASH (Magoc and Salzberg, 2011) with the parameter that overlap was longer than 10 bp and its mismatch rate was lower than 0.2. Paired-end reads were then sorted by sample-specific barcodes and clustered into operational taxonomic units (OTUs) using Uparse (vsesion 7.1, http://drive5.com/uparse/) at 97% identity. The phylogenetic classification was analyzed by RDP Classifier (http://rdp. cme.msu.edu/) with confidence threshold of 70% based on the Silva (SSU r138) database. Then sequences belonged to “o__Chloroplast” at Order level were removed, and randomly resampled to the depth of minimum number of sample sequences. Alpha-diversity index (sobs and shannon) was were calculated based on mothur (version v.1.30.1, http://www.mothur.org/wiki/Schloss_SOP#Alpha_diversity). Non-metric multidimensional scaling (NMDS) and analysis of similarity (ANOSIM) were elucidated at OUTs level based on bray-curtis distance, using Canoco 5 (Šmilauer and Lepš, 2014) and *Vegan* package in R, respectively.

Sequences associated with nitrogen fixation (*nifH*) and denitrification (*nirK*) were filtered, merged and clustered on Majorbio Cloud Platform with the same parameters as 16S rRNA gene sequences. The top 90% (relative abundance) OTUs in each sanple were selected and searched against NCBI (https://blast.ncbi.nlm.nih.gov/Blast.cgi) using Blastn. The closest matches found in the NCBI database were included for phylogenetic analysis. All OTUs and reference sequences were aligned using MAFFT (http://align.bmr.kyushu-u.ac.jp/mafft/online/server/), and then cured using Gblock (http://www.phylogeny.fr/one_task.cgi?task_type=gblocks). The phylogenetic tree was constructed with MEGA7 (Kumar et al., 2016) using the Neighbor-Joining method based on 1000 replicates.

**Management of shotgun metagenome sequencing data**

The metagenomic library (insert size 350-480 bp) was prepared using a TruseqTM DNA Sample Prep Kit (Illumina, San Diego, CA, USA), then sequenced using Illumina platforms (Supplementary Table S2). Raw sequence data quality was assessed using FastQC (http://www.bioinformatics.babraham.ac.uk/projects/fastqc/), trimmed using Seqprep (https://github.com/jstjohn/SeqPrep), and then high quality reads were extracted by filtering low quality reads with ‘N’ base, reads<Q20, and short reads<50 bp using Sikle (https://github.com/najoshi/sickle). We preformed *de novo* assembly upon de Brujin graph approach using a multiple mixing assembly strategy. Firstly, the cleaned sequence reads were assembled into contigs using IDBA-UD (Peng et al., 2012)(k-mer=47-97). Secondly, aligning clean reads to the contigs (>200 bp) using bowite 2 (Langmead and Salzberg, 2012), and then the reads unmapped were assembled again using IDBA-UD. Finaly, the contigs <1000 bp above were assembled again using Newbler (https://ngs.csr.uky.edu/Newbler), and all contigs above were pooled together. In light of some low-abundance microbes that were not detected in the limited sequencing data, we combined all the contigs assembled from natural biocrusts reads in 2015, 2016 and 2017 to build a non-redundant gene catalogue, while the two batches of natural biocrusts in 2018 build non-redundant gene catalogue separately. A non-redundant gene catalogue for simulated biocrusts metagenomic analyses was obtained with all simulated biocrusts contigs. The contigs (≥300 bp) were applied for Open reading frames (ORFs) prediction using MetaGene (Noguchi et al., 2006). To construct a non-redundant gene catalog, pairwise comparison of predicted ORFs (≥100 bp) was performed using CD-HIT (Li and Godzik, 2006) at 95% identity and 90% coverage. All high-quality reads were aligned (95% identity) against gene catalog by SOAPaligner (Li et al., 2008) to obtain the gene abundance in each sample. The abundance of each unigene was normalized with gene length as previously described (Qin et al., 2012; Zheng et al., 2016). First of all, a raw count matrix is inferred from copy numbers of the reads mapped to a gene divided by the length of the corresponding gene. Then the relative gene abundance profile is calculated using the gene abundance information from the raw count matrix divided by the sum of the abundance of the entire gene that could be classified in the database. Taxonomic classification was conducted by searching the putative amino acid sequences, which translated from the gene catalogue, against the NR databases (June 2018) using BLASTp (BLAST Version 2.3.0; E-value ≤1e-5). To analyze the nitrogen cycle involved by microorganisms, and minimize the influence of bryophytes whose genome size is much larger than microbes, the sequences in the classification result that are "Bryopsida" at the class level are removed from analysis. The remaining sequences are used for subsequent functional annotations using BLASTp (BLAST Version 2.3.0; E-value ≤1e-5) against KEGG (version 59, Kyoto Encyclopedia of Genes and Genomes) database (Kanehisa and Goto, 1999), and then normalized the abundance of each KO (KEGG Ortholog) to 1 000 000. The corresponding relationship between all the N-cycle associated genes and KO number is shown in Supplementary Table S3. To calculate and compare the relative abundance of the nitrogen transformation pathways, each gene combination or pathway was summed (multiple pathways performing the same conversion step) or averaged (multiple enzymes/subunits in the same conversion step) (Llorens-Mares et al., 2015). To compare with the data obtained from other methods such as GeoChip and qPCR, as well as compare with other studies, marker genes of N-cycling were determined according to literature research and used for the analysis of microbial taxa, co-occurrence network and environmental factors influence.

**Statistical analysis**

The significant differences between various indicators in the samples were determined with SPSS 20.0 software (IBM, USA) using one-way analysis of variance (ANOVA) with Tukey’s test at p<0.05. Differences in bacterial community composition and N cycling among different stages of biocrusts were examined with Non-metric multidimensional scaling analysis (NMDS). Environmental factors that shaped nitrogen cycle significantly (p<0.05) on pathway level or gene level for natural biocrusts were showed with forward selection of redundancy analysis (RDA). NMDS and RDA were conducted using Canoco 5 (Šmilauer and Lepš, 2014). Analysis of similarity (ANOSIM) were conducted to test the statistical significance of differences between different types of biocrusts with *vegan* package in R, performing 10 000 permutations. The relative contribution of the microbial taxa related to each key gene of N cycling was calculated according to the annotation in KO and taxa of each unigene. We merged the genus abundance profile of the five batches, and selected the high abundance genus (average abundance for each type in each batch above 0.01%, represent the top 833 of all 5998 detected genus) for the network analysis. Spearman’s correlations between N-cycling key genes and high abundance genus, between N-transformation pathways, as well as between environmental factors and N-transformation pathways, were calculated using *psych* package in R 3.4.4. All network diagrams were visualized with Cytoscape (Shannon et al., 2003). The co-occurrence network of the N-cycling key genes and high abundance genus was clustered with an edge-weighted spring-embedded algorithm with correlation coefficient r score. Each connection stands for a significant (p<0.001) correlation. ModuLand as a Cytoscape plug-in (Kovacs et al., 2010; Szalay-Beko et al., 2012) was used for network modularization, with a threshold of merging modules at 0.9. The network dissimilarity (β_w_) between different successional stages was determined using previously described methods (Koleff et al., 2003; Liu et al., 2019).

**Supplementary References**

Bates, S.T., Nash, T.H., Sweat, K.G., and Garcia-Pichel, F. (2010). Fungal communities of lichen-dominated biological soil crusts: Diversity, relative microbial biomass, and their relationship to disturbance and crust cover. *Journal of Arid Environments* 74(10)**,** 1192-1199. doi: 10.1016/j.jaridenv.2010.05.033.

Cadillo-Quiroz, H., Brauer, S., Yashiro, E., Sun, C., Yavitt, J., and Zinder, S. (2006). Vertical profiles of methanogenesis and methanogens in two contrasting acidic peatlands in central New York State, USA. *Environmental Microbiology* 8(8)**,** 1428-1440. doi: 10.1111/j.1462-2920.2006.01036.x.

Fierer, N., Jackson, J.A., Vilgalys, R., and Jackson, R.B. (2005). Assessment of soil microbial community structure by use of taxon-specific quantitative PCR assays. *Appl Environ Microbiol* 71(7)**,** 4117-4120. doi: 10.1128/AEM.71.7.4117-4120.2005.

Kanehisa, M., and Goto, S. (1999). KEGG: Kyoto encyclopedia of genes and genomes. *Nucleic Acids Research* 28(1)**,** 27-30.

Koleff, P., Gaston, K.J., and Lennon, J.J. (2003). Measuring beta diversity for presence-absence data. *Journal of Animal Ecology* 72(3)**,** 367-382. doi: DOI 10.1046/j.1365-2656.2003.00710.x.

Kovacs, I.A., Palotai, R., Szalay, M.S., and Csermely, P. (2010). Community landscapes: an integrative approach to determine overlapping network module hierarchy, identify key nodes and predict network dynamics. *PLoS One* 5(9)**,** e12528. doi: 10.1371/journal.pone.0012528.

Kumar, S., Stecher, G., and Tamura, K. (2016). MEGA7: Molecular Evolutionary Genetics Analysis Version 7.0 for Bigger Datasets. *Mol Biol Evol* 33(7)**,** 1870-1874. doi: 10.1093/molbev/msw054.

Langmead, B., and Salzberg, S.L. (2012). Fast gapped-read alignment with Bowtie 2. *Nat Methods* 9(4)**,** 357-359. doi: 10.1038/nmeth.1923.

Li, R.Q., Li, Y.R., Kristiansen, K., and Wang, J. (2008). SOAP: short oligonucleotide alignment program. *Bioinformatics* 24(5)**,** 713-714. doi: 10.1093/bioinformatics/btn025.

Li, W.Z., and Godzik, A. (2006). Cd-hit: a fast program for clustering and comparing large sets of protein or nucleotide sequences. *Bioinformatics* 22(13)**,** 1658-1659. doi: 10.1093/bioinformatics/btl158.

Liu, L., Chen, H., Liu, M., Yang, J.R., Xiao, P., Wilkinson, D.M., et al. (2019). Response of the eukaryotic plankton community to the cyanobacterial biomass cycle over 6 years in two subtropical reservoirs. *ISME J* 13(9)**,** 2196-2208. doi: 10.1038/s41396-019-0417-9.

Llorens-Mares, T., Yooseph, S., Goll, J., Hoffman, J., Vila-Costa, M., Borrego, C.M., et al. (2015). Connecting biodiversity and potential functional role in modern euxinic environments by microbial metagenomics. *ISME J* 9(7)**,** 1648-1661. doi: 10.1038/ismej.2014.254.

Magoc, T., and Salzberg, S.L. (2011). FLASH: fast length adjustment of short reads to improve genome assemblies. *Bioinformatics* 27(21)**,** 2957-2963. doi: 10.1093/bioinformatics/btr507.

Noguchi, H., Park, J., and Takagi, T. (2006). MetaGene: prokaryotic gene finding from environmental genome shotgun sequences. *Nucleic Acids Research* 34(19)**,** 5623-5630. doi: 10.1093/nar/gkl723.

Nubel, U., Garcia-Pichel, F., and Muyzer, G. (1997). PCR primers to amplify 16S rRNA genes from cyanobacteria. *Appl Environ Microbiol* 63(8)**,** 3327-3332. doi: 10.1128/aem.63.8.3327-3332.1997.

Peng, Y., Leung, H.C., Yiu, S.M., and Chin, F.Y. (2012). IDBA-UD: a de novo assembler for single-cell and metagenomic sequencing data with highly uneven depth. *Bioinformatics* 28**,** 1420–1428. doi: 10.1093/bioinformatics/bts174.

Qin, J.J., Li, Y.R., Cai, Z.M., Li, S.H., Zhu, J.F., Zhang, F., et al. (2012). A metagenome-wide association study of gut microbiota in type 2 diabetes. *Nature* 490(7418)**,** 55-60. doi: 10.1038/nature11450.

Rosch, C., Mergel, A., and Bothe, H. (2002). Biodiversity of Denitrifying and Dinitrogen-Fixing Bacteria in an Acid Forest Soil. *Applied and Environmental Microbiology* 68(8)**,** 3818-3829. doi: 10.1128/aem.68.8.3818-3829.2002.

Shannon, P., Markiel, A., Ozier, O., Baliga, N.S., Wang, J.T., Ramage, D., et al. (2003). Cytoscape: A software environment for integrated models of biomolecular interaction networks. *Genome Research* 13(11)**,** 2498-2504. doi: 10.1101/gr.1239303.

Šmilauer, P., and Lepš, J. (2014). *Multivariate analysis of ecological data using CANOCO 5.* Cambridge: Cambridge University Press.

Szalay-Beko, M., Palotai, R., Szappanos, B., Kovacs, I.A., Papp, B., and Csermely, P. (2012). ModuLand plug-in for Cytoscape: determination of hierarchical layers of overlapping network modules and community centrality. *Bioinformatics* 28(16)**,** 2202-2204. doi: 10.1093/bioinformatics/bts352.

Wu, D., Horn, M.A., Behrendt, T., Muller, S., Li, J., Cole, J.A., et al. (2019). Soil HONO emissions at high moisture content are driven by microbial nitrate reduction to nitrite: tackling the HONO puzzle. *ISME J* 13(7)**,** 1688-1699. doi: 10.1038/s41396-019-0379-y.

Zheng, Z.J., Zhong, W.D., Liu, L., Wu, C.Y., Zhang, L.S., Cai, S.F., et al. (2016). Bioinformatics approaches for human gut microbiome research. *Infectious Diseases and Translational Medicine* 2(2)**,** 69-79. doi: 10.11979/idtm.201602005.
